# The Concise Guide to PHARMACOLOGY 2025/26: Catalytic receptors

**DOI:** 10.1111/bph.70233

**Published:** 2025-12

**Authors:** Stephen P. H. Alexander, Doriano Fabbro, Chloe J. Peach, Alasdair J. Gibb, Eamonn Kelly, Alistair A. Mathie, Emma L. Veale, Jane F. Armstrong, Elena Faccenda, Simon D. Harding, Christopher Southan, Jamie A. Davies, Annie Beuve, Peter Brouckaert, Clare Bryant, John C. Burnett, Richard W. Farndale, Andreas Friebe, John Garthwaite, Adrian J. Hobbs, Gavin E. Jarvis, Laura Kilpatrick, Doris Koesling, Michaela Kuhn, Birgit Leitinger, David MacEwan, Tom P. Monie, Lincoln R. Potter, Michael Russwurm, Harald H. H. W. Schmidt, Johannes-Peter Stasch, Scott A. Waldman

**Affiliations:** 1 Division of Physiology, Pharmacology & Neuroscience, School of Life Sciences, University of Nottingham Medical School, Nottingham, NG7 2UH, UK; 2 PIQUR Therapeutics, Basel, 4057, Switzerland; 3 Division of Physiology, Pharmacology & Neuroscience, School of Life Sciences, Centre of Membrane Proteins and Receptors (COMPARE), University of Nottingham Medical School, Nottingham, NG7 2UH, UK; 4 Research Department of Neuroscience, Physiology and Pharmacology, Division of Biosciences, University College London, Gower Street, London, WC1E 6BT, UK; 5 School of Psychology and Neuroscience, University of Bristol, Bristol, BS8 1TD, UK; 6 School of Life Sciences, University of Westminster, London, W1W 6UW, UK; 7 Medway School of Pharmacy, Universities of Kent and Greenwich, Chatham Maritime, Kent, ME4 4TB, UK; 8 Institute for Neuroscience and Cardiovascular Research, University of Edinburgh, Edinburgh, EH8 9XD, UK; 9 New Jersey Medical School at Rutgers, New Jersey, USA; 10 Ghent University, Ghent, Belgium; 11 University of Cambridge, Cambridge, UK; 12 Mayo Clinic, Rochester, USA; 13 University of Wurzburg, Wurzburg, Germany; 14 University College London, London, UK; 15 Queen Mary University of London, London, UK; 16 University of Sunderland, Sunderland, UK; 17 University of Nottingham, Nottingham, UK; 18 Ruhr University Bochum, Bochum, Germany; 19 University of Wurzburg, Wurzburg, Germany; 20 Imperial College, London, UK; 21 University of Liverpool, Liverpool, UK; 22 University of Minnesota, Minneapolis, USA; 23 Maastricht University, Maastricht, The Netherlands; 24 Bayer HealthCare, Wuppertal, Germany; 25 Thomas Jefferson University, Philadelphia, USA

## Abstract

The Concise Guide to Pharmacology 2025/26 marks the seventh edition in this series of biennial publications in the British Journal of Pharmacology. Presented in landscape format, the guide provides a comparative overview of the pharmacology of drug target families. The concise nature of the Concise Guide refers to the style of presentation, being clear, accessible, and well-structured, rather than the scope of the content, which spans approximately 500 pages. The Concise Guide summarises the key pharmacological properties of around 1900 human drug targets, and nearly 7000 interactions, involving around 4400 ligands. While the content is a substantially condensed version of the more detailed information and links available at the www.guidetopharmacology.org website, the printed guide serves as a permanent, citable, point-in-time record, that remains stable despite ongoing updates to the online database. The full contents of this publication can be found at https://bpspubs.onlinelibrary.wiley.com/doi/10.1111/bph.70233.

The Concise Guides provide expert-curated recommendations of ‘Gold Standard’ selective pharmacological tools, available either commercially or as donations, which enable the identification of individual drug targets or families of drug targets. While the Concise Guide offers a more streamlined overview, more comprehensive information, including detailed pharmacological profiles and links to multiple online databases, is available through the Guide to Pharmacology website. The 2025/26 edition of the Concise Guide is based on material current as of mid-2025, and supersedes all previous editions, including the 2023/24 Guide, and earlier Guides to Receptors and Channels. It is produced in close conjunction with the Nomenclature and Standards Committee of the International Union of Basic and Clinical Pharmacology (NC-IUPHAR), and as such provides official IUPHAR classification and nomenclature for human drug targets, where applicable.

Catalytic receptors are one of the six major pharmacological targets into which the Guide is divided, with the others being: G protein-coupled receptors, ion channels, nuclear hormone receptors, enzymes and transporters. Each section includes nomenclature guidance, concise summaries, information of the best available pharmacological tools, key references, and suggestions for further reading.

## Graphical Abstract

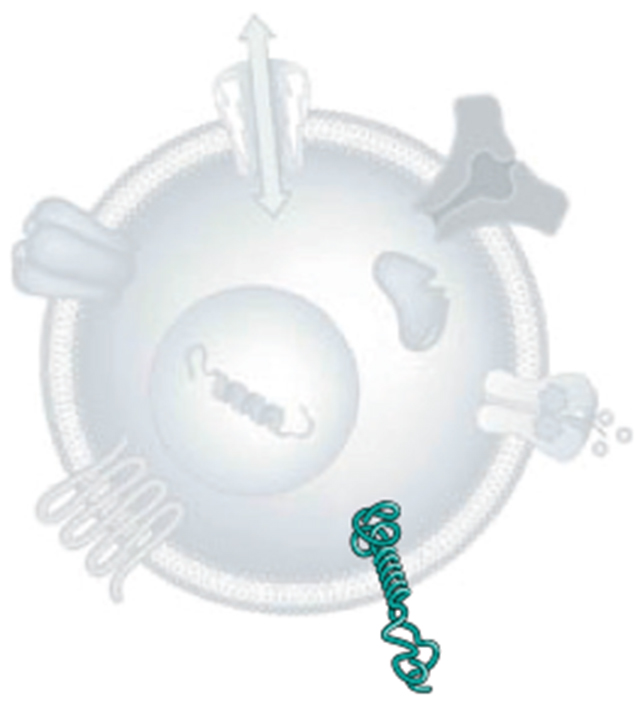

## Introduction:

Catalytic receptors are cell-surface proteins, usually dimeric in nature, which encompass ligand binding and functional domains in one polypeptide chain. The ligand binding domain is placed on the extracellular surface of the plasma membrane and separated from the functional domain by a single transmembrane-spanning domain of 20-25 hydrophobic amino acids. The functional domain on the intracellular face of the plasma membrane has catalytic activity, or interacts with particular enzymes, giving the superfamily of receptors its name. Endogenous agonists of the catalytic receptor superfamily are peptides or proteins, the binding of which may induce dimerization of the receptor, which is the functional version of the receptor.

Amongst the catalytic receptors, particular subfamilies may be readily identified dependent on the function of the enzymatic portion of the receptor. The smallest group is the particulate guanylyl cyclases of the natriuretic peptide receptor family. The most widely recognized group is probably the receptor tyrosine kinase (RTK) family, epitomized by the neurotrophin receptor family, where a crucial initial step is the activation of a signalling cascade by autophosphorylation of the receptor on intracellular tyrosine residue(s) catalyzed by enzyme activity intrinsic to the receptor. A third group is the extrinsic protein tyrosine kinase receptors, where the catalytic activity resides in a separate protein from the binding site. Examples of this group include the GDNF and ErbB receptor families, where one, catalytically silent, member of the heterodimer is activated upon binding the ligand, causing the second member of the heterodimer, lacking ligand binding capacity, to initiate signaling through tyrosine phosphorylation. A fourth group, the receptor threonine/serine kinase (RTSK) family, exemplified by TGF-β and BMP receptors, has intrinsic serine/threonine protein kinase activity in the heterodimeric functional unit. A fifth group is the receptor tyrosine phosphatases (RTP), which appear to lack cognate ligands, but may be triggered by events such as cell:cell contact and have identified roles in the skeletal, hematopoietic and immune systems.

A further group of catalytic receptors for the Guide is the integrins, which have roles in cell:cell communication, often associated with signaling in the blood.

## Cytokine receptor family

Catalytic receptors → Cytokine receptor family

### Overview:

Cytokines are not a clearly defined group of agents, other than having an impact on immune signalling pathways, although many cytokines have effects on other systems, such as in development. A feature of some cytokines, which allows them to be distinguished from hormones, is that they may be produced by “non-secretory” cells, for example, endothelial cells. Within the cytokine receptor family, some subfamilies may be identified, which are described elsewhere in the Guide to PHARMACOLOGY, receptors for the TNF family, the TGF-β family and the chemokines. Within this group of records are described Type I cytokine receptors, typified by interleukin receptors, and Type II cytokine receptors, exemplified by interferon receptors. These receptors possess a conserved extracellular region, known as the cytokine receptor homology domain (CHD), along with a range of other structural modules, including extracellular immunoglobulin (Ig)-like and fibronectin type III (FBNIII)-like domains, a transmembrane domain, and intracellular homology domains. An unusual feature of this group of agents is the existence of soluble and decoy receptors. These bind cytokines without allowing signalling to occur. A further attribute is the production of endogenous antagonist molecules, which bind to the receptors selectively and prevent signalling. A commonality of these families of receptors is the ligand-induced homo- or hetero-oligomerisation, which results in the recruitment of intracellular protein partners to evoke cellular responses, particularly in inflammatory or haematopoietic signalling. Although not an exclusive signalling pathway, a common feature of the majority of cytokine receptors is activation of the JAK/STAT pathway. This cascade is based around the protein tyrosine kinase activity of the Janus kinases (JAK), which phosphorylate the receptor and thereby facilitate the recruitment of signal transducers and activators of transcription (STATs). The activated homo- or heterodimeric STATs function principally as transcription factors in the nucleus.

**Type I cytokine receptors** are characterized by two pairs of conserved cysteines linked via disulfide bonds and a C-terminal WSXWS motif within their CHD. Type I receptors are commonly classified into five groups, based on sequence and structual homology of the receptor and its cytokine ligand, which is potentially more reflective of evolutionary relationships than an earlier scheme based on the use of common signal transducing chains within a receptor complex.

**Type II cytokine receptors** also have two pairs of conserved cysteines but with a different arrangement to Type I and also lack the WSXWS motif.

## IL-2 receptor family

Catalytic receptors → Cytokine receptor family → IL-2 receptor family

### Overview:

The IL-2 receptor family consists of one or more ligand-selective subunits, and a common γ chain (γc): *IL2RG*, P31785), though IL-4 and IL-7 receptors can form complexes with other receptor chains. Receptors of this family associate with Jak1 and Jak3, primarily activating Stat5, although certain family members can also activate Stat1, Stat3, or Stat6. Ro264550 has been described as a selective IL-2 receptor antagonist, which binds to IL-2 [261].

### Complexes

**Table:** 

Nomenclature	Interleukin-2 receptor	Interleukin-4 receptor type I	Interleukin-4 receptor type II	Interleukin-7 receptor
Subunits	Interleukin-2 receptor subunit α (Ligand-binding subunit), Interleukin-2 receptor subunit β (Ligand-binding subunit), Interleukin-2 receptor subunit γ (Other subunit)	Interleukin-4 receptor subunit α (Ligand-binding subunit), Interleukin-2 receptor subunit γ (Other subunit)	Interleukin-4 receptor subunit α (Ligand-binding subunit), Interleukin-13 receptor subunit α1 (Other subunit)	Interleukin-7 receptor subunit α (Ligand-binding subunit), Interleukin-2 receptor subunit γ (Other subunit)
Endogenous agonists	IL-2 (*IL2*, P60568)	IL-4 (*IL4*, P05112)	IL-13 (*IL13*, P35225), IL-4 (*IL4*, P05112)	IL-7 (*IL7*, P13232)
Endogenous antagonists	IL-1 receptor antagonist (*IL1RN*, P18510)	–	–	–
Selective antagonists	AF12198 [1]	–	–	–

**Table:** 

Nomenclature	Interleukin-9 receptor	Interleukin-15 receptor	Interleukin-21 receptor	Thymic stromal lymphopoietin receptor
Subunits	Interleukin 9 receptor (Ligand-binding subunit), Interleukin-2 receptor subunit γ (Other subunit)	Interleukin-2 receptor subunit β (Ligand-binding subunit), Interleukin-15 receptor subunit α (Ligand-binding subunit), Interleukin-2 receptor subunit γ (Other subunit)	Interleukin 21 receptor (Ligand-binding subunit), Interleukin-2 receptor subunit γ (Other subunit)	Interleukin-7 receptor subunit α (Ligand-binding subunit), Cytokine receptor-like factor 2 (Other subunit)
Endogenous agonists	IL-9 (*IL9*, P15248)	IL-15 (*IL15*, P40933) [308]	IL-21 (*IL21*, Q9HBE4)	thymic stromal lymphopoietin (*TSLP*, Q969D9)

### Subunits

**Table:** 

Nomenclature	Interleukin 13 receptor, α2	Interleukin-2 receptor subunit α	Interleukin-2 receptor subunit β	Interleukin-2 receptor subunit γ	Interleukin-4 receptor subunit α
HGNC, UniProt	*IL13RA2*, Q14627	*IL2RA*, P01589	*IL2RB*, P14784	*IL2RG*, P31785	*IL4R*, P24394
Antibodies	–	daclizumab (Binding) (p*K*_d_>8) [223], basiliximab (Binding)	–	–	dupilumab (Binding) (pIC_50_ 11.1) [182]
Comments	Decoy receptor that binds IL-13 (*IL13*, P35225) as a monomer.	–	–	–	–

**Table:** 

Nomenclature	Interleukin-7 receptor subunit α	Interleukin 9 receptor	Interleukin-13 receptor subunit α1	Interleukin-15 receptor subunit α	Interleukin 21 receptor	Cytokine receptor-like factor 2
HGNC, UniProt	*IL7R*, P16871	*IL9R*, Q01113	*IL13RA1*, P78552	*IL15RA*, Q13261	*IL21R*, Q9HBE5	*CRLF2*, Q9HC73

Further reading on IL-2 receptor familyCagdasD
 (2021) Genomic Spectrum and Phenotypic Heterogeneity of Human IL-21 Receptor Deficiency. J Clin Immunol
41: 1272–129033929673
10.1007/s10875-021-01031-5PMC8086229LeonardWJ
 (2019) The γ_c_ Family of Cytokines: Basic Biology to Therapeutic Ramifications.Immunity
50: 832–85030995502
10.1016/j.immuni.2019.03.028

## IL-3 receptor family

Catalytic receptors → Cytokine receptor family → IL-3 receptor family

### Overview:

The IL-3 receptor family signal through a receptor complex comprising of a ligand-specific α subunit and a common β chain (*CSF2RB*, P32927), which is associated with Jak2 and signals primarily through Stat5.

### Complexes

**Table:** 

Nomenclature	Interleukin-3 receptor	Interleukin-5 receptor	Granulocyte macrophage colony-stimulating factor receptor
Subunits	Interleukin 3 receptor, α subunit (Ligand-binding subunit), Cytokine receptor common β subunit (Other subunit)	Interleukin 5 receptor, α subunit (Ligand-binding subunit), Cytokine receptor common β subunit (Other subunit)	GM-CSF receptor, α subunit (Ligand-binding subunit), Cytokine receptor common β subunit (Other subunit)
Endogenous agonists Selective antagonists	IL-3 (*IL3*, P08700)	IL-5 (*IL5*, P05113)	G-CSF (*CSF3*, P09919), GM-CSF (*CSF2*, P04141)
Selective antagonists	–	YM90709 [195]	–

### Subunits

**Table:** 

Nomenclature	Interleukin 3 receptor, α subunit	Interleukin 5 receptor, α subunit	GM-CSF receptor, α subunit	Cytokine receptor common β subunit
HGNC, UniProt	*IL3RA*, P26951	*IL5RA*, Q01344	*CSF2RA*, P15509	*CSF2RB*, P32927
Endogenous agonists	IL-3 (*IL3*, P08700)	IL-5 (*IL5*, P05113)	GM-CSF (*CSF2*, P04141)	–
Antibodies	–	benralizumab (Binding) (p*K*_d_ 8.7) [144]	mavrilimumab (Binding) (pIC_50_ 9.9) [41]	–

## IL-6 receptor family

Catalytic receptors → Cytokine receptor family → IL-6 receptor family

### Overview:

The IL-6 receptor family signal through a ternary receptor complex consisting of the cognate receptor and either the IL-6 signal transducer gp130 (*IL6ST*, P40189) or the oncostatin M-specific receptor, β subunit (*OSMR*, Q99650), which then activates the JAK/STAT, Ras/Raf/MAPK and PI 3-kinase/PKB signalling modules. Unusually amongst the cytokine receptors, the CNTF receptor is a glycophosphatidylinositol-linked protein.

### Complexes

**Table:** 

Nomenclature	Interleukin-6 receptor	Interleukin-11 receptor	Interleukin-27 receptor	Interleukin-31 receptor	Ciliary neutrophic factor receptor	Leukemia inhibitory factor receptor	Oncostatin-M receptor
Subunits	Interleukin-6 receptor, α subunit (Ligand-binding subunit), Interleukin-6 receptor, β subunit (Other subunit)	Interleukin-11 receptor, α subunit (Ligand-binding subunit), Interleukin-6 receptor, β subunit (Other subunit)	Interleukin-6 receptor, β subunit (Other subunit), Interleukin 27 receptor, alpha (Ligand-binding subunit)	Interleukin-31 receptor, α subunit (Ligand-binding subunit), Oncostatin M-specific receptor, β subunit (Other subunit)	Ciliary neurotrophic factor receptor α subunit (Ligand-binding subunit), Leukemia inhibitory factor receptor (Other subunit), Interleukin-6 receptor, β subunit	Leukemia inhibitory factor receptor (Ligand-binding subunit), Interleukin-6 receptor, β subunit (Other subunit)	Oncostatin M-specific receptor, β subunit (Ligand-binding subunit), Interleukin-6 receptor, β subunit (Other subunit)
Endogenous agonists	IL-6 (*IL6*, P05231) (Murine NIH/3T3 fibroblasts with human IL6R exhibited a single class of binding sites for 125I-labeled recombinant human interleukin-6 (125I-rhIL-6) (Kd = 440 pM, 20,000 receptors per cell).) [227]	IL-11 (*IL11*, P20809)	IL-27 (*EBI3IL27*, Q14213Q8NEV9)	IL-31 (*IL31*, Q6EBC2)	CRCF1/CLCF1 heterodimer (*CLCF1*CRLF1, O75462Q9UBD9), ciliary neurotrophic factor (*CNTF*, P26441)	LIF (*LIF*, P15018), cardiotrophin-1 (*CTF1*, Q16619), oncostatin M (*OSM*, P13725)	oncostatin M (*OSM*, P13725)
Agonists	–	oprelvekin [15, 284]	–	–	–	–	–
Antibodies	vobarilizumab (Binding) (p*K*_d_ 12.7) [272], satralizumab (Binding) (p*K*_d_ 8.9) [123], tocilizumab (Binding) (p*K*_d_ 8.6)	–	–	–	–	–	–

### Subunits

**Table:** 

Nomenclature	Interleukin-6 receptor, α subunit	Interleukin-6 receptor, β subunit
Systematic nomenclature	interleukin 6 receptor	interleukin 6 signal transducer
Common abbreviation	IL6R	IL6ST
HGNC, UniProt	*IL6R*, P08887	*IL6ST* P40189
Endogenous agonists	IL-6 (*IL6*, P05231) (IL6R) expressed stably in murine NIH/3T3 fibroblasts.exhibited a single class of binding sites for 125I-labeled recombinant human interleukin-6 (125I-rhIL-6) (Kd = 440 pM, 20,000 receptors per cell).) [227]	–
Antibodies	sarilumab (Binding) (p*K*_d_ 10.6–11.1) [254]	–

**Table:** 

Nomenclature	Interleukin-11 receptor, α subunit	Interleukin 27 receptor, alpha	Interleukin-31 receptor, α subunit	Ciliary neurotrophic factor receptor α subunit	Leptin receptor	Leukemia inhibitory factor receptor	Oncostatin M-specific receptor, β subunit
HGNC, UniProt	*IL11RA*, Q14626	*IL27RA*, Q6UWB1	*IL31RA*, Q8NI17	*CNTFR*, P26992	*LEPR*, P48357	*LIFR*, P42702	*OSMR*, Q99650
Agonists	–	–	–	–	leptin (*LEP*, P41159) [273] – Mouse	–	–

Further reading on IL-6 receptor familyHoLJ
 (2015) Biological effects of interleukin-6: Clinical applications in autoimmune diseases and cancers. Biochem Pharmacol
97: 16–2626080005
10.1016/j.bcp.2015.06.009KangS
 (2019) Targeting Interleukin-6 Signaling in Clinic. Immunity
50: 1007–102330995492
10.1016/j.immuni.2019.03.026MurakamiM
 (2019) Pleiotropy and Specificity: Insights from the Interleukin 6 Family of Cytokines. Immunity
50: 812–83130995501
10.1016/j.immuni.2019.03.027RothaugM
 (2016) The role of interleukin-6 signaling in nervous tissue. Biochim Biophys Acta
1863: 1218–2727016501
10.1016/j.bbamcr.2016.03.018

## IL-12 receptor family

Catalytic receptors → Cytokine receptor family → IL-12 receptor family

### Overview:

IL-12 receptors are a subfamily of the IL-6 receptor family. IL12RB1 is shared between receptors for IL-12 and IL-23; the functional agonist at IL-12 receptors is a heterodimer of IL-12A/IL-12B, while that for IL-23 receptors is a heterodimer of IL-12B/IL-23A.

### Complexes

**Table:** 

Nomenclature	Interleukin-12 receptor	Interleukin-23 receptor
Subunits	Interleukin-12 receptor, β1 subunit (Ligand-binding subunit), Interleukin-12 receptor, β2 subunit (Other subunit)	Interleukin-12 receptor, β1 subunit (Ligand-binding subunit), Interleukin 23 receptor (Ligand-binding subunit)
Inhibitors	–	icotrokinra (p*K*_d_ 11.7) [73], icotrokinra (pIC_50_ 11.3) [73]
Endogenous agonists	IL-12 (*IL12A*IL12B, P29459P29460)	IL-23 (*IL12B*IL23A, P29460) [152]

### Subunits

**Table:** 

Nomenclature	Interleukin-12 receptor, β1 subunit	Interleukin-12 receptor, β2 subunit	Interleukin 23 receptor
HGNC, UniProt	*IL12RB1*, P42701	*IL12RB2*, Q99665	*IL23R*, Q5VWK5

Further reading on IL-12 receptor familyTait WojnoED
 (2019) The Immunobiology of the Interleukin-12 Family: Room for Discovery. Immunity
50: 851–87030995503
10.1016/j.immuni.2019.03.011PMC6472917

## Prolactin receptor family

Catalytic receptors → Cytokine receptor family → Prolactin receptor family

### Overview:

Prolactin family receptors form homodimers in the presence of their respective ligands, associate exclusively with Jak2 and signal via Stat5.

**Table:** 

Nomenclature	Eythropoietin receptor	Granulocyte colony-stimulating factor receptor	Growth hormone receptor	Prolactin receptor	Thrombopoietin receptor
HGNC, UniProt	*EPOR*, P19235	*CSF3R*, Q99062	*GHR*, P10912	*PRLR*, P16471	*MPL*, P40238
Endogenous agonists	erythropoietin (*EPO*, P01588) [67]	G-CSF (*CSF3*, P09919)	growth hormone 1 (*GH1*, P01241) [112], growth hormone 2 (*GH2*, P01242)	choriomammotropin (*CSH1CSH2*, P0DML2P0DML3), chorionic somatomammotropin hormone-like 1 (*CSHL1*, Q14406)	thrombopoietin (*THPO*, P40225)
Agonists	peginesatide [67]	pegfilgrastim	–	prolactin (*PRL*, P01236) [75] – Mouse	hetrombopag, romiplostim
Selective agonists	–	–	–	–	eltrombopag [179]
Antagonists	–	–	pegvisomant [265]	–	–

Further reading on Prolactin receptor familyCabrera-ReyesEA
 (2017) Prolactin function and putative expression in the brain. Endocrine
57: 199–21328634745
10.1007/s12020-017-1346-xGoffinV. (2017) Prolactin receptor targeting in breast and prostate cancers: New insights into an old challenge. Pharmacol Ther
179: 111–12628549597
10.1016/j.pharmthera.2017.05.009

## Interferon receptor family

Catalytic receptors → Cytokine receptor family → Interferon receptor family

### Overview:

The interferon receptor family includes receptors for type I (α, βκ and ω) and type II (γ) interferons. There are at least 13 different genes encoding IFN-α subunits in a cluster on human chromosome 9p22: α1 (*IFNA1*, P01562), α2 (*IFNA2*, P01563), α4 (*IFNA4*, P05014), α5 (*IFNA5*, P01569), α6 (*IFNA6*, P05013), α7 (*IFNA7*, P01567), α8 (*IFNA8*, P32881), α10 (*IFNA10*, P01566), α13 (*IFNA13*, P01562), α14 (*IFNA14*, P01570), α16 (*IFNA16*, P05015), α17 (*IFNA17*, P01571) and α21 (*IFNA21*, P01568).

### Complexes

**Table:** 

Nomenclature	Interferon-α/β receptor	Interferon-γ receptor
Subunits	Interferon α/β receptor 2 (Other subunit), interferon α/β receptor 1 (Ligand-binding subunit)	Interferon γ receptor 1 (Ligand-binding subunit), Interferon γ receptor 2 (Other subunit)
Endogenous agonists	IFN-α1/13 (*IFNA1*IFNA13, P01562), IFN-α10 (*IFNA10*, P01566), IFN-α14 (*IFNA14*, P01570), IFN-α16 (*IFNA16*, P05015), IFN-α17 (*IFNA17*, P01571), IFN-α2 (*IFNA2*, P01563), IFN-α21 (*IFNA21*, P01568), IFN-α4 (*IFNA4*, P05014), IFN-α5 (*IFNA5*, P01569), IFN-α6 (*IFNA6*, P05013), IFN-α7 (*IFNA7*, P01567), IFN-α8 (*IFNA8*, P32881), IFN-β (*IFNB1*, P01574), IFN-κ (*IFNK*, Q9P0W0), IFN-ω (*IFNW1*, P05000)	IFN-γ (*IFNG*, P01579)
Selective agonists	peginterferon alfa-2b [278]	–

### Subunits

**Table:** 

Nomenclature	interferon α/β receptor 1	Interferon α/β receptor 2	Interferon γ receptor 1	Interferon γ receptor 2
HGNC, UniProt	*IFNAR1*, P17181	*IFNAR2*, P48551	*IFNGR1*, P15260	*IFNGR2*, P38484
Endogenous agonists	IFN-β (*IFNB1*, P01574) [302]	–	–	–
Selective agonists	peginterferon alfa-2b [278]	–	–	–
Antibodies	anifrolumab (Binding) (p*K*_d_>10) [33]	–	–	–

Further reading on Interferon receptor familyKotenkoSV
 (2017) Contribution of type III interferons to antiviral immunity: location, location, location. J Biol Chem
292: 7295–730328289095
10.1074/jbc.R117.777102PMC5418032LazearHM
 (2019) Shared and Distinct Functions of Type I and Type III Interferons. Immunity
50: 907–92330995506
10.1016/j.immuni.2019.03.025PMC6839410NgCT
 (2016) Alpha and Beta Type 1 Interferon Signaling: Passage for Diverse Biologic Outcomes. Cell
164: 349–5226824652
10.1016/j.cell.2015.12.027PMC4733246SchreiberG. (2017) The molecular basis for differential type I interferon signaling. J Biol Chem
292: 7285–729428289098
10.1074/jbc.R116.774562PMC5418031

## IL-10 receptor family

Catalytic receptors → Cytokine receptor family → IL-10 receptor family

### Overview:

The IL-10 family of receptors are heterodimeric combinations of family members: IL10RA/IL10RB responds to IL-10; IL20RA/IL20RB responds to IL-19, IL-20 and IL-24; IL22RA1/IL20RB responds to IL-20 and IL-24; IL22RA1/IL10RB responds to IL-22; IFNLR1(previouly known as IL28RA)/IL10RB responds to IFN-λ1, -λ2 and -λ3 (previouly known as IL-29, IL-28A and IL-28B respectively).

### Complexes

**Table:** 

Nomenclature	Interleukin-10 receptor	Interleukin-20 receptor	Interleukin-22α1/20β heteromer	Interleukin-22α1/10β heteromer	Interferon-λ receptor 1
Subunits	Interleukin 10 receptor, α subunit (Ligand-binding subunit), Interleukin 10 receptor, β subunit (Other subunit)	Interleukin 20 receptor, α subunit (Ligand-binding subunit), Interleukin 20 receptor, β subunit (Other subunit)	Interleukin 20 receptor, β subunit (Ligand-binding subunit), Interleukin 22 receptor, α1 subunit (Ligand-binding subunit)	Interleukin 10 receptor, β subunit (Ligand-binding subunit), Interleukin 22 receptor, α1 subunit (Ligand-binding subunit)	Interleukin 10 receptor, β subunit (Other subunit), Interferon-λ receptor subunit 1 (Ligand-binding subunit)
Endogenous agonists	IL-10 (*IL10*, P22301)	IL-19 (*IL19*, Q9UHD0), IL-20 (*IL20*, Q9NYY1), IL-24 (*IL24*, Q13007)	IL-20 (*IL20*, Q9NYY1), IL-24 (*IL24*, Q13007)	IL-22 (*IL22*, Q9GZX6) [253]	IFN-λ1 (*IFNL1*, Q8IU54), IFN-λ2 (*IFNL2*, Q8IZJ0), IFN-λ3 (*IFNL3*, Q8IZI9)

### Subunits

**Table:** 

Nomenclature	Interleukin-22 receptor α2	Interleukin 10 receptor, α subunit	Interleukin 10 receptor, β subunit	Interleukin 20 receptor, α subunit	Interleukin 20 receptor, β subunit	Interleukin 22 receptor, α1 subunit	Interferon-λ, receptor subunit 1
HGNC, UniProt	*IL22RA2*, Q969J5	*IL10RA*, Q13651	*IL10RB*, Q08334	*IL20RA*, Q9UHF4	*IL20RB*, Q6UXL0	*IL22RA1*, Q8N6P7	*IFNLR1*, Q8IU57
Comments	Soluble decoy receptor that binds IL-22 (*IL22*, Q9GZX6) as a monomer.	–	–	–	–	–	–

Further reading on IL-10 receptor familyFelixJ
 (2017) Mechanisms of immunomodulation by mammalian and viral decoy receptors: insights from structures. Nat Rev Immunol
17: 112–12928028310
10.1038/nri.2016.134OuyangW
 (2019) IL-10 Family Cytokines IL-10 and IL-22: from Basic Science to Clinical Translation. Immunity
50: 871–89130995504
10.1016/j.immuni.2019.03.020

## Immunoglobulin-like family of IL-1 receptors

Catalytic receptors → Cytokine receptor family → Immunoglobulin-like family of IL-1 receptors

### Overview:

The immunoglobulin-like family of IL-1 receptors are heterodimeric receptors made up of a cognate receptor subunit and an IL-1 receptor accessory protein, *IL1RAP* (Q9NPH3, also known as C3orf13, IL-1RAcP, IL1R3). They are characterised by extracellular immunoglobulin-like domains and an intracellular Toll/Interleukin-1R (TIR) domain.

### Complexes

**Table:** 

Nomenclature	Interleukin-1 receptor, type I	Interleukin-33 receptor	Interleukin-36 receptor	Interleukin-1 receptor, type II	Interleukin-18 receptor
Subunits	Interleukin 1 receptor, type I (Ligand-binding subunit), IL-1 receptor accessory protein (Other subunit)	Interleukin-1 receptor-like 1 (Ligand-binding subunit), IL-1 receptor accessory protein (Other subunit)	Interleukin-1 receptor-like 2 (Ligand-binding subunit), IL-1 receptor accessory protein (Other subunit)	IL-1 receptor accessory protein (Other subunit), Interleukin 1 receptor, type II (Ligand-binding subunit)	Interleukin-18 receptor 1 (Ligand-binding subunit), IL-18 receptor accessory protein (Other subunit)
Inhibitors	anakinra (p*K*_d_ 7.8) [60]	–	–	–	–
Endogenous agonists	IL-1α (*IL1A*, P01583), IL-1β (*IL1B*, P01584)	IL-33 (*IL33*, O95760)	IL-36α (*IL36A*, Q9UHA7), IL-36α (*IL36B*, Q9NZH7), IL-36γ (*IL36G*, Q9NZH8)		IL-18 (*IL18*, Q14116), IL-37 (*IL37*, Q9NZH6)
Endogenous antagonists	IL-1 receptor antagonist (*IL1RN*, P18510)	–	IL-36 receptor antagonist (*IL36RN*, Q9UBH0)	–	–
Selective antagonists	AF12198 [1]	–	–	–	–
Comments	–	–	IL-36 receptor antagonist (*IL36RN*, Q9UBH0) is a highly specific antagonist of the response to IL-36γ (*IL36G*, Q9NZH8).	Decoy receptor that binds IL-1α (*IL1A*, P01583), IL-1β (*IL1B*, P01584) and IL-1 receptor antagonist (*IL1RN*, P18510).	–

### Subunits

**Table:** 

Nomenclature	Interleukin 1 receptor, type I	Interleukin 1 receptor, type II	Interleukin-1 receptor-like 1	Interleukin-1 receptor-like 2	Interleukin-18 receptor 1
HGNC, UniProt	*IL1R1*, P14778	*IL1R2*, P27930	*IL1RL1*, Q01638	*IL1RL2*, Q9HB29	*IL18R1*, Q13478

Further reading on Immunoglobulin-like family of IL-1 receptorsAfoninaIS
 (2015) Proteolytic Processing of Interleukin-1 Family Cytokines: Variations Common Theme. Immunity
42: 991–100426084020
10.1016/j.immuni.2015.06.003MantovaniA
 (2019) Interleukin-1 and Related Cytokines in the Regulation of Inflammation and Immunity. Immunity
50: 778–79530995499
10.1016/j.immuni.2019.03.012PMC7174020

## IL-17 receptor family

Catalytic receptors → Cytokine receptor family → IL-17 receptor family

### Overview:

The IL17 cytokine family consists of six ligands (IL-17A-F), which signal through five receptors (IL-17RA-E).

### Complexes

**Table:** 

Nomenclature	Interleukin-17 receptor	Interleukin-25 receptor	Interleukin-17C receptor
Subunits	Interleukin 17 receptor A (Ligand-binding subunit), interleukin 17 receptor C (Other subunit)	Interleukin 17 receptor A (Other subunit), Interleukin 17 receptor B (Ligand-binding subunit)	Interleukin 17 receptor A (Other subunit), Interleukin 17 receptor E (Ligand-binding subunit)
Endogenous agonists	IL-17A (*IL17A*, Q16552), IL-17A/IL-17F (*IL17A*IL17F, Q16552Q96PD4), IL-17F (*IL17F*, Q96PD4)	IL-17B (*IL17B*, Q9UHF5), IL-25 (*IL25*, Q9H293)	IL-17C (*IL17C*, Q9P0M4)

### Subunits

**Table:** 

Nomenclature	Interleukin 17 receptor A	Interleukin 17 receptor B	interleukin 17 receptor C	Interleukin-17 receptor D	Interleukin 17 receptor E
HGNC, UniProt	*IL17RA*, Q96F46	*IL17RB*, Q9NRM6	*IL17RC*, Q8NAC3	*IL17RD*, Q8NFM7	*IL17RE*, Q8NFR9
Antibodies	brodalumab (Binding) (p*K*_d_ 9.2) [264]	–	–	–	–
Comments	–	–	–	The endogenous agonist for this receptor is unknown.	–

Further reading on IL-17 receptor familyBeringerA
 (2016) IL-17 in Chronic Inflammation: From Discovery to Targeting. Trends Mol Med
22: 230–24126837266
10.1016/j.molmed.2016.01.001LubbertsE. (2015) The IL-23-IL-17 axis in inflammatory arthritis. Nat Rev Rheumatol
11: 415–2925907700
10.1038/nrrheum.2015.53McGeachyMJ
 (2019) The IL-17 Family of Cytokines in Health and Disease. Immunity
50: 892–90630995505
10.1016/j.immuni.2019.03.021PMC6474359

## GDNF Family Receptor (GFR)

Catalytic receptors → GDNF Family Receptor (GFR)

### Overview:

GDNF family receptors (GFR) are extrinsic co-receptors, where ligand binding to the extracellular domain of the glycosylphosphatidylinositol-linked cell-surface GFRs activates a transmembrane tyrosine kinase enzyme, RET. The endogenous ligands are typically dimeric, linked through disulphide bridges: glial cell-derived neurotrophic factor GDNF (*GDNF*, P39905) (211 aa); neurturin (*NRTN*, Q99748) (197 aa); artemin (*ARTN*, Q5T4W7) (237 aa) and persephin (*PSPN*, O60542) (156 aa), referred to as GDNF family ligands (GFLs). There is evidence for RET-dependent and RET-independent signalling [122]. Growth/Differentiation Factor 15 (GDF15) has been shown to activate GFRAL, a transmembrane protein that similarly forms a complex with RET [197, 295].

**Table:** 

Nomenclature	GDNF family receptor α1	GDNF family receptor α2	GDNF family receptor α3	GDNF family receptor α4
Common abbreviation	GFRα1	GFRα2	GFRα3	GFRα4
HGNC, UniProt	*GFRA1*, P56159	*GFRA2* O00451	*GFRA3*, O60609	*GFRA4*, Q9GZZ7
Potency order	GDNF (*GDNF*, P39905) > neurturin (*NRTN*, Q99748) > artemin (*ARTN*, Q5T4W7)	neurturin (*NRTN*, Q99748) > GDNF (*GDNF*, P39905)	artemin (*ARTN*, Q5T4W7)	persephin (*PSPN*, O60542)
Labelled ligands	[^125^I]GDNF (rat) (pK_d_ 10.2–11.5) [142, 266]	–	–	–

Further reading on GDNF Family Receptor (GFR)AllenSJ
 (2013) GDNF, NGF and BDNF as therapeutic options for neurodegeneration. Pharmacol Ther
138: 155–7523348013
10.1016/j.pharmthera.2013.01.004IbáñezCF
 (2017) Biology of GDNF and its receptors - Relevance for disorders of the central nervous system. Neurobiol Dis
97: 80–8926829643
10.1016/j.nbd.2016.01.021MerighiA. (2016) Targeting the glial-derived neurotrophic factor and related molecules for controlling normal and pathologic pain. Expert Opin Ther Targets
20: 193–20826863504
10.1517/14728222.2016.1085972

## Integrins

Catalytic receptors → Integrins

### Overview:

Integrins are unusual signalling proteins that function to signal both from the extracellular environment into the cell, but also from the cytoplasm to the external of the cell. The intracellular signalling cascades associated with integrin activation focus on protein kinase activities, such as focal adhesion kinase and Src. Based on this association between extracellular signals and intracellular protein kinase activity, we have chosen to include integrins in the ‘Catalytic receptors’ section of the database until more stringent criteria from NC-IUPHAR allows precise definition of their classification. Integrins are heterodimeric entities, composed of α and β subunits, each 1TM proteins, which bind components of the extracellular matrix or counter-receptors expressed on other cells. One class of integrin contains an inserted domain (I) in its α subunit, and if present (in α1, α2, α10, α11, αD, αE, αL, αM and αX), this I domain contains the ligand binding site. All β subunits possess a similar I-like domain, which has the capacity to bind ligand, often recognising the RGD motif. The presence of an α subunit I domain precludes ligand binding through the β subunit. Integrins provide a link between ligand and the actin cytoskeleton (through typically short intracellular domains). Integrins bind several divalent cations, including a Mg^2+^ ion in the I or I-like domain that is essential for ligand binding. Other cation binding sites may regulate integrin activity or stabilise the 3D structure. Integrins regulate the activity of particular protein kinases, including focal adhesion kinase and integrin-linked kinase. Cellular activation regulates integrin ligand affinity *via* inside-out signalling and ligand binding to integrins can regulate cellular activity *via* outside-in signalling. Several drugs that target integrins are in clinical use including: (1) tirofiban (αIIbβ3) for short term prevention of coronary thrombosis (abciximab, the first clinically approved (1994) chimeric monoclonal antibody, is no longer available for clinical use), (2) vedolizumab (α4β7) to reduce gastrointestinal inflammation, and (3) natalizumab (α4β1) in some cases of severe multiple sclerosis. Drugs targeting multiple integrins are under investigation for the treatment of dry eye disease, *e.g.*, risuteganib, an anti-integrin peptide [242].

### Complexes

**Table:** 

Nomenclature	integrin α1β1	integrin α2β1	integrin αIIbβ3	integrin α4β1
Subunits	integrin, alpha 1 subunit, integrin, beta 1 subunit (fibronectin receptor, beta polypeptide, antigen CD29 includes MDF2, MSK12)	integrin, alpha 2 subunit (CD49B, alpha 2 subunit of VLA-2 receptor), integrin, beta 1 subunit (fibronectin receptor, beta polypeptide, antigen CD29 includes MDF2, MSK12)	integrin, alpha IIb subunit (platelet glycoprotein IIb of IIb/IIIa complex, antigen CD41), integrin, beta 3 subunit (platelet glycoprotein IIIa, antigen CD61)	integrin, alpha 4 subunit (antigen CD49D, alpha 4 subunit of VLA-4 receptor), integrin, beta 1 subunit (fibronectin receptor, beta polypeptide, antigen CD29 includes MDF2, MSK12)
Ligands	collagen, laminin	collagen, laminin, thrombospondin	fibrinogen (*FGA*FGBFGG, P02671P02675P02679), fibronectin (*FN1*, P02751), von Willebrand factor (*VWF*, P04275), vitronectin (*VTN*, P04004), thrombospondin	fibronectin (*FN1*, P02751), vascular cell adhesion protein 1 (*VCAM1*, P19320), osteopontin (*SPP1*, P10451), thrombospondin
Inhibitors	obtustatin (pIC_50_ 9.1) [178]	TCI15 (pIC_50_ 7.9) [191], BTT-3033 [204]	tirofiban (pIC_50_ 9.4) [267], G4120 (p*K*_i_ 8.4) [186, 297], GR 144053 (pIC_50_ 7.4) [63], eptifibatide (pIC_50_ 6.2–6.8) [234]	BIO1211 (pIC_50_ 8.3–9) [162], carotegrast (pIC_50_ 7.2) [176], TCS2314, firategrast [95], valategrast [113], zaurategrast [288]
Antagonists	–	–	–	BOP (p*K*_d_ 11.3) [218]
Antibodies	–	–	abciximab (Binding) [43]	natalizumab (Inhibition) [205]
Comments	–	–	–	LDV-FITC is used as a probe at this receptor.

**Table:** 

Nomenclature	integrin α4β7	integrin α5β1	integrin α6β1	integrin α10β1
Subunits	integrin, alpha 4 subunit (antigen CD49D, alpha 4 subunit of VLA-4 receptor), integrin, beta 7 subunit	integrin, alpha 5 subunit (fibronectin receptor, alpha polypeptide), integrin, beta 1 subunit (fibronectin receptor, beta polypeptide, antigen CD29 includes MDF2, MSK12)	integrin, alpha 6 subunit, integrin, beta 1 subunit (fibronectin receptor, beta polypeptide, antigen CD29 includes MDF2, MSK12)	integrin, alpha 10 subunit, integrin, beta 1 subunit (fibronectin receptor, beta polypeptide, antigen CD29 includes MDF2, MSK12)
Endogenous ligands	–	cellular communication network factor 3 (*CCN3*, P48745) (Binding) (p*K*_d_ 7.3) [161]	–	–
Ligands	–	fibronectin (*FN1*, P02751)	laminin	collagen
Inhibitors	emvistegrast (pIC_50_ 10.3) [19], carotegrast (pIC_50_ 8.5) [176], firategrast [95], valategrast [l13], zaurategrast [288]	risuteganib [18]		
Selective inhibitors	etrolizumab (Binding) (pIC_50_ 10.1) [71]	–	–	–
Antibodies	vedolizumab (Antagonist) (pIC_50_ 8.3) [221]	volociximab (Binding) (p*K*_d_ 9.5) [16, 17]	–	–

**Table:** 

Nomenclature	integrin α11β1	integrin αEβ7	integrin αLβ2	integrin αVβ3
Subunits	integrin, alpha 11 subunit, integrin, beta 1 subunit (fibronectin receptor, beta polypeptide, antigen CD29 includes MDF2, MSK12)	integrin, alpha E subunit (antigen CD103, human mucosal lymphocyte antigen 1; alpha polypeptide), integrin, beta 7 subunit	integrin, alpha L subunit (antigen CD11A (p180), lymphocyte function-associated antigen 1; alpha polypeptide), integrin, beta 2 subunit (complement component 3 receptor 3 and 4 subunit)	integrin, alpha V subunit, integrin, beta 3 subunit (platelet glycoprotein IIIa, antigen CD61)
Endogenous ligands	–	–	–	cellular communication network factor 3 (*CCN3*, P48745) (Binding) (p*K*_d_ 8) [161]
Ligands	collagen	E-cadherin	ICAM-1 (*ICAM1*, P05362), ICAM-2 (*ICAM2*, P13598)	vitronectin (*VTN*, P04004), fibronectin (*FN1*, P02751), fibrinogen (*FGA*FGBFGG, P02671P02675P02679), osteopontin (*SPP1*, P10451), von Willebrand factor (*VWF*, P04275), thrombospondin, tenascin
Activators	–	–	–	TP508 (p*K*_d_ 7.9) [55]
Inhibitors	–	–	A286982 (pIC_50_ 7.4–7.5) [166]	risuteganib
Antagonists	–	–	–	echistatin (pIC_50_ 11.7) [155], P11 (pIC_50_ 11.6) [155], CWHM12 (pIC_50_ 9.1) [111], cilengitide (pIC_50_ 8.5) [92], GSK2603566A (pIC_50_ 8.4) [4]
Antibodies	–	etrolizumab (Inhibition) [252]	–	etaracizumab (Binding) (p*K*_d_ 6.3) [291]

### Subunits

**Table:** 

Nomenclature	integrin, alpha 1 subunit	integrin, alpha 2 subunit (CD49B, alpha 2 subunit of VLA-2 receptor)	integrin, alpha IIb subunit (platelet glycoprotein IIb of IIb/IlIa complex, antigen CD41)	integrin, alpha 3 subunit (antigen CD49C, alpha 3 subunit of VLA-3 receptor)	integrin, alpha 4 subunit (antigen CD49D, alpha 4 subunit of VLA-4 receptor)	integrin, alpha 5 subunit (fibronectin receptor, alpha polypeptide)
HGNC, UniProt	*ITGA1*, P56199	*ITGA2*, P17301	*ITGA2B*, P08514	*ITGA3*, P26006	*ITGA4*, P13612	*ITGA5*, P08648
Ligands	–	–	–	peptide ligand 2 (Binding) (pIC_50_ 7.2) [296]	–	–
Inhibitors	–	–	–	–	carotegrast, carotegrast methyl [78], firategrast [95], valategrast [113], zaurategrast [288]	–
Antibodies	–	–	–	–	abrilumab (Inhibition) (p*K*_d_ 11.1) [214], natalizumab (Inhibition) [205]	volociximab (Binding) (p*K*_d_ 9.5) [16, 17]

**Table:** 

Nomenclature	integrin, alpha 6 subunit	integrin, alpha 7 subunit	integrin, alpha 8 subunit	integrin, alpha 9 subunit	integrin, alpha 10 subunit	integrin, alpha 11 subunit	integrin, alpha D subunit
HGNC, UniProt	*ITGA6*, P23229	*ITGA7* Q13683	*ITGA8*, P53708	*ITGA9*, Q13797	*ITGA10*, O75578	*ITGA11*, Q9UKX5	*ITGAD*, Q13349
Endogenous ligands	–	–	–	SVEP1 (*SVEP1*, Q4LDE5) (Binding) [232]	–	–	–
Antagonists	–	–	–	BOP (p*K*_d_ 11.5) [218]	–	–	–

**Table:** 

Nomenclature	integrin, alpha E subunit (antigen CD103, human mucosal lymphocyte antigen 1; alpha polypeptide)	integrin, alpha L subunit (antigen CD11A (p180), lymphocyte function-associated antigen 1; alpha polypeptide)	integrin, alpha M subunit (complement component 3 receptor 3 subunit)	integrin, alpha V subunit	integrin, alpha X subunit (complement component 3 receptor 4 subunit)	integrin, beta 1 subunit (fibronectin receptor, beta polypeptide, antigen CD29 includes MDF2, MSK12)
HGNC, UniProt	*ITGAE*, P38570	*ITGAL*, P20701	*ITGAM*, P11215	*ITGAV*, P06756	*ITGAX*, P20702	*ITGB1*, P05556
Antagonists	–	lifitegrast (Inhibition) [24, 304]	–	MK-0429 (pIC_50_ 7.1) [120]	–	–
Antibodies	–	efalizumab (Binding) (p*K*_d_ 11.4) [127]	–	–	–	–

**Table:** 

Nomenclature	integrin, beta 2 subunit (complement component 3 receptor 3 and 4 subunit)	integrin, beta 3 subunit (platelet glycoprotein IlIa, antigen CD61)	integrin, beta 4 subunit	integrin, beta 5 subunit	integrin, beta 6 subunit	integrin, beta 7 subunit	integrin, beta 8 subunit
HGNC, UniProt	*ITGB2*, P05107	*ITGB3*, P05106	*ITGB4*, P16144	*ITGB5*, P18084	*ITGB6*, P18564	*ITGB7* P26010	*ITGB8*, P26012
Inhibitors	–	compound 7 (p*K*_i_ 6.8) [12]	–	compound 7 (p*K*_i_ 7.3) [12]	–	–	compound 7 (p*K*_i_ 7.6) [12]
Selective inhibitors	–	–	–	–	compound 7 (p*K*_i_ 10) [12]	–	–
Antibodies	–	–	–	–	–	etrolizumab (Binding) [252]	–

### Comments: Integrin ligands

Collagen is the most abundant protein in metazoa, rich in glycine and proline residues, made up of cross-linked triple helical structures, generated primarily by fibroblasts. Extensive post-translational processing is conducted by prolyl and lysyl hydroxylases, as well as transglutaminases. Over 40 genes for collagen-α subunits have been identified in the human genome. The collagen-binding integrins α1β1, α2β1, α10β1 and α11β1 recognise a range of triple-helical peptide motifs including GFOGER (O = hydroxyproline), a synthetic peptide derived from the primary sequence of collagen I (COL1A1 (*COL1A1*, P02452)) and collagen II (COL2A1 (*COL2A1*, P02458)). Related motifs have been have identified in many other collagens.

**Laminin** is an extracellular glycoprotein composed of α, β and γ chains, for which five, four and three genes, respectively, are identified in the human genome. It binds to α1β1, α2β1, α3,β1, α7β1 and α6β4 integrins.

**Fibrinogen** (***FGA*** P02671, ***FGB*** P02675, ***FGC*** P02679) is a glycosylated hexamer composed of two α (*FGA*, P02671), two β (*FGB*, P02675) and two γ (*FGG*, P02679) subunits, linked by disulphide bridges. It is found in plasma and alpha granules of platelets. It forms cross-links between activated platelets mediating aggregation by binding αIIbβ3; proteolysis by thrombin cleaves short peptides termed fibrinopeptides to generate fibrin, which polymerises as part of the blood coagulation cascade.

**Fibronectin** (***FN1***, **P02751**) is a disulphide-linked homodimer found as two major forms; a soluble dimeric form found in the plasma and a tissue version that is polymeric, which is secreted into the extracellular matrix by fibroblasts. Splice variation of the gene product (*FN1*, P02751) generates multiple isoforms.

**Vitronectin** (***VTN***, **P04004**) is a serum glycoprotein and extracellular matrix protein which is found either as a monomer or, following proteolysis, a disulphide -linked dimer.

**Osteopontin** (***SPP1***, **P10451**) forms an integral part of the mineralized matrix in bone, where it undergoes extensive post-translation processing, including proteolysis and phosphorylation.

**von Willebrand factor** (***VWF***, **P04275**) is a glycoprotein synthesised in vascular endothelial cells as a disulphide-linked homodimer, but multimerises further in plasma and is deposited on vessel wall collagen as a high molecular weight multimer. It is responsible for capturing platelets under arterial shear flow (*via* GPIb) and in thrombus propagation (via integrin αIIbβ3).

Further reading on IntegrinsClemetsonKJ. (2017) The origins of major platelet receptor nomenclature. Platelets
28: 40–4227715379
10.1080/09537104.2016.1204437EmsleyJ
 (2000) Structural basis of collagen recognition by integrin alpha2beta1. Cell
101: 47–5610778855
10.1016/S0092-8674(00)80622-4HamaiaS
 (2014) Integrin recognition motifs in the human collagens. Adv Exp Med Biol
819: 127–4225023172
10.1007/978-94-017-9153-3_9HamidiH
 (2016) The complexity of integrins in cancer and new scopes for therapeutic targeting. Br J Cancer
115: 1017–102327685444
10.1038/bjc.2016.312PMC5117799HortonER
 (2016) The integrin adhesome network at a glance. J Cell Sci
129: 4159–416327799358
10.1242/jcs.192054PMC5117201ManninenA
 (2017) A proteomics view on integrin-mediated adhesions. Proteomics
17:10.1002/pmic.20160002227723259SlackRJ
 (2022) Emerging therapeutic opportunities for integrin inhibitors. Nat Rev Drug Discov
21: 60–7834535788
10.1038/s41573-021-00284-4PMC8446727

## Pattern recognition receptors

Catalytic receptors → Pattern recognition receptors

### Overview:

Pattern Recognition Receptors (PRRs, [258]) (nomenclature as agreed by **NC-IUPHAR sub-committee on Pattern Recognition Receptors**, [28]) participate in the innate immune response to microbial agents, the stimulation of which leads to activation of intracellular enzymes and regulation of gene transcription. PRRs express multiple leucine-rich regions to bind a range of microbially-derived ligands, termed PAMPs or pathogen-associated molecular patterns or endogenous ligands, termed DAMPS or damage-associated molecular patterns. These include peptides, carbohydrates, peptidoglycans, lipoproteins, lipopolysaccharides, and nucleic acids. PRRs include both cell-surface and intracellular proteins. PRRs may be divided into signalling-associated members, identified here, and endocytic members, the function of which appears to be to recognise particular microbial motifs for subsequent cell attachment, internalisation and destruction. Some are involved in inflammasome formation, and modulation of IL-1β cleavage and secretion, and others in the initiation of the type I interferon response.

PRRs included in the Guide To PHARMACOLOGY database but not the Concise Guide are:

### Catalytic PRRs

Caspase 4 and caspase 5

### Non-catalytic PRRs

Absent in melanoma (AIM)-like receptors (ALRs)

C-type lectin-like receptors (CLRs)

Other pattern recognition receptors

Advanced glycosylation end-product specific receptor (RAGE)

## Toll-like receptor family

Catalytic receptors → Pattern recognition receptors → Toll-like receptor family

### Overview:

Members of the toll-like family of receptors (nomenclature recommended by the NC-IUPHAR subcommittee on pattern recognition receptors, [28]) share significant homology with the interleukin-1 receptor family and appear to require dimerization either as homo- or heterodimers for functional activity. Heterodimerization appears to influence the potency of ligand binding substantially (*e.g.* TLR1/2 and TLR2/6, [259, 260]). TLR1, TLR2, TLR4, TLR5, TLR6 and TLR11 are cell-surface proteins, while other members are associated with intracellular organelles, signalling through the MyD88-dependent pathways (with the exception of TLR3). As well as responding to exogenous infectious agents [69], it has been suggested that selected members of the family may be activated by endogenous ligands, such as hsp60 (*HSPD1*, P10809) [206].

**Table:** 

Nomenclature	TLR1	TLR2	TLR3	TLR4	TLR5
HGNC, UniProt	*TLR1*, Q15399	*TLR2*, O60603	*TLR3*, O15455	*TLR4*, O00206	*TLR5*, O60602
Agonists	–	compound 13 [121], peptidoglycan [238, 299]	poly(I:C) [3]	LPS [220], paclitaxel [135] – Mouse	flagellin [105]
Selective antagonists	–	–	–	resatorvid [124]	–
Comments	Functions as a heterodimer with TLR2 in detection of triacylated lipoproteins. Activated by the synthetic analogue Pam3CSK4.	Functions as a heterodimer with either TLR1 or TLR6 in the detection of triacylated and diacylated lipopeptides respectively. TLR1/2 and 2/6 heterodimers can be activated by the synthetic lipopeptides Pam3CSK4 and Pam2CSK4 respectively. There is some debate in the field as to whether or not peptidoglycan is a direct agonist of TLR2, or whether the early studies reporting this contained contaminating lipoproteins.	Involved in endosomal detection of dsRNA; pro-inflammatory.	Eritoran (E5564) is a lipid A analogue, which has been described as a TLR4 antagonist [126]. TLR4 signals in conjunction with the co-factor MD-2 (LY96).	Involved in the detection of bacterial flagellin; pro-inflammatory.

**Table:** 

Nomenclature	TLR6	TLR7	TLR8	TLR9	TLR10	TLR11
HGNC, UniProt	*TLR6*, Q9Y2C9	*TLR7* Q9NYK1	*TLR8*, Q9NR97	*TLR9*, Q9NR96	*TLR10*, Q9BXR5	–
Agonists	–	resiquimod [109, 132, 151], imiquimod [151], loxoribine [107]	resiquimod [109, 132, 151]	–	–	–
Antagonists	–	hydroxychloroquine (pIC_50_ 5.6) [150]	–	hydroxychloroquine (pIC_50_ 7.1) [150]	–	–
Comments	Functions as a heterodimer with TLR2. Involved in the pro-inflammatory response to diacylated bacterial lipopeptides.	Activated by imidazoquinoline derivatives and RNA oligoribonucleotides. Involved in endosomal detection of ssRNA; pro-inflammatory.	Activated by imidazoquinoline derivatives and RNA oligoribonucleotides. Endosomal detection of ssRNA; pro-inflammatory.	Toll-like receptor 9 interacts with unmethylated CpG dinucleotides from bacterial DNA [110]. Activated by CpG rich DNA sequences; pro-inflammatory.	TLR10 is the only pattern-recognition receptor without known ligand specificity and biological function. Evidence suggests it plays a modulatory role with predominantly inhibitory (anti-inflammatory) actions [212]. Murine TLR10 has a retroviral insertion that makes it nonfunctional.	Found in mouse

Further reading on Toll-like receptor familyAnthoneyN
 (2018) Toll and Toll-like receptor signalling in development. Development
145:10.1242/dev.15601829695493BryantCE
 (2015) International Union of Basic and Clinical Pharmacology. XCVI. Pattern recognition receptors in health and disease. Pharmacol Rev
67: 462–50425829385
10.1124/pr.114.009928PMC4394686FranzKM
 (2017) Innate Immune Receptors as Competitive Determinants of Cell Fate. Mol Cell
66: 750–76028622520
10.1016/j.molcel.2017.05.009PMC5503483JoostenLA
 (2016) Toll-like receptors and chronic inflammation in rheumatic diseases: new developments. Nat Rev Rheumatol
12: 344–5727170508
10.1038/nrrheum.2016.61NunesKP
 (2019) Targeting toll-like receptor 4 signalling pathways: can therapeutics pay the toll for hypertension?
Br J Pharmacol
176: 1864–187929981161
10.1111/bph.14438PMC6534778ZhangZ
 (2017) Toward a structural understanding of nucleic acid-sensing Toll-like receptors in the innate immune system. FEBS Lett
591: 3167–318128686285
10.1002/1873-3468.12749

## NOD-like receptor family

Catalytic receptors → Pattern recognition receptors → NOD-like receptor family

### Overview:

The nucleotide-binding oligomerization domain, leucine-rich repeat (NLR) family of receptors (nomenclature recommended by the NC-IUPHAR subcommittee on pattern recognition receptors [28]) share a common domain organisation. This consists of an N-terminal effector domain, a central nucleotide-binding and oligomerization domain (NOD; also referred to as a NACHT domain), and C-terminal leucine-rich repeats (LRR) which have regulatory and ligand recognition functions. The type of effector domain has resulted in the division of NLR family members into two major sub-families, NLRC and NLRP, along with three smaller sub-families NLRA, NLRB and NLRX [262]. NLRC members express an N-terminal caspase recruitment domain (CARD) and NLRP members an N-terminal Pyrin domain (PYD).

Upon activation the NLRC family members NOD1 (NLRC1) and NOD2 (NLRC2) recruit a serine/threonine kinase *RIPK2* (receptor interacting serine/threonine kinase 2, O43353, also known as CARD3, CARDIAK, RICK, RIP2) leading to signalling through NFκB and MAP kinase. Activation of NLRC4 (previously known as IPAF) and members of the NLRP3 family, including NLRP1 and NLRP3, leads to formation of a large multiprotein complex known as the inflammasome. In addition to NLR proteins other key members of the inflammasome include the adaptor protein ASC (apoptosis-associated speck-like protein containing a CARD, also known as *PYCARD*, CARD5, TMS1, Q9ULZ3) and inflammatory caspases. The inflammasome activates the pro-inflammatory cytokines IL-1β (*IL1B*, P01584) and IL-18 (*IL18*, Q14116) [28, 50].

**Table:** 

Nomenclature	nucleotide binding oligomerization domain containing 1	nucleotide binding oligomerization domain containing 2	NLRC3	NLRC4	NLRC5	NLRX1	CIITA
Common abbreviation	NOD1	NOD2	–	–	–	–	–
HGNC, UniProt	*NOD1*, Q9Y239	*NOD2*, Q9HC29	*NLRC3*, Q7RTR2	*NLRC4*, Q9NPP4	*NLRC5*, Q86WI3	*NLRX1*, Q86UT6	*CIITA*, P33076
Agonists	meso-DAP	muramyl dipeptide	–	–	–	–	–
Comments	–	NOD2 has also been reported to be activated by ssRNA [230] although this has not been widely reproduced.		NLRC4 forms an inflammasome with the NAIP proteins following recognition of bacterial flagellin and type III secretion system rod proteins by the NAIPs.	–	–	–

**Table:** 

Nomenclature	NLRP1	NLRP2	NLRP3	NLRP4	NLRP5	NLRP6	NLRP7
HGNC, UniProt	*NLRP1*, Q9C000	*NLRP2*, Q9NX02	*NLRP3*, Q96P20	*NLRP4*, Q96MN2	*NLRP5*, P59047	*NLRP6*, P59044	*NLRP7* Q8WX94
Inhibitors	–	–	MCC950 (pIC_50_>8) [42]	–	–	–	–
Agonists	muramyl dipeptide	–	–	–	–	–	–
Comments	NLRP1 has 3 murine orthologues which lack the N-terminal Pyrin domain. Murine NLRPlb (ENSMUSG00000070390) is the best characterised, responding to Anthrax Lethal Toxin.	Along with NLRP7, NLRP2 is the product of a primate-specific gene duplication.	NLRP3 has been shown to be activated following disruption of cellular haemostasis by a wide-variety of exogenous and endogenous molecules. The identity of the precise agonist that interacts with NLRP3 remains enigmatic. Efflux of potassium ions appears to be a common event for NLRP3 activating molecules. In addition to MCC950 [42] other small molecules including CY-09 [129], β-hydroxybutyrate [300], and various boron containing compounds [11] modulate NLRP3.	Expanded in the mouse resulting in 7 orthologues.	–	–	Absent in mouse. Along with NLRP2 the product of a primate-specific gene duplication.

**Table:** 

Nomenclature	NLRP8	NLRP9	NLRP10	NLRP11	NLRP12	NLRP13	NLRP14
HGNC, UniProt	*NLRP8*, Q86W28	*NLRP9*, Q7RTR0	*NLRP10*, Q86W26	*NLRP11*, P59045	*NLRP12*, P59046	*NLRP13*, Q86W25	*NLRP14*, Q86W24
Comments	Absent in mouse	This receptor has three murine orthologues.	NLRP10 lacks the LRR region.	Absent in mouse	–	Absent in mouse	–

### Comments:

NLRP3 has also been reported to respond to host-derived products, known as danger-associated molecular patterns, or DAMPs, including uric acid [183], ATP, L-glucose, hyaluronan and amyloid β (*APP*, P05067) [236].

Loss-of-function mutations of NLRP3 are associated with cold autoinflammatory and Muckle-Wells syndromes.

This family also includes NLR family, apoptosis inhibitory protein (*NAIP*, Q13075) which can be found in the ‘Inhibitors of apoptosis (IAP) protein family’ in the Other protein targets section of the Guide.

Further reading on NOD-like receptor familyBrozP
 (2016) Inflammasomes: mechanism of assembly, regulation and signalling. Nat Rev Immunol
16: 407–2027291964
10.1038/nri.2016.58BryantCE
 (2015) International Union of Basic and Clinical Pharmacology. XCVI. Pattern recognition receptors in health and disease. Pharmacol Rev
67: 462–50425829385
10.1124/pr.114.009928PMC4394686Keestra-GounderAM
 (2017) NOD1 and NOD2: Beyond Peptidoglycan Sensing. Trends Immunol
38: 758–76728823510
10.1016/j.it.2017.07.004PMC5624830Lei-LestonAC
 (2017) Epithelial Cell Inflammasomes in Intestinal Immunity and Inflammation. Front Immunol
8: 116828979266
10.3389/fimmu.2017.01168PMC5611393ManSM. (2018) Inflammasomes in the gastrointestinal tract: infection, cancer and gut microbiota homeostasis. Nat Rev Gastroenterol Hepatol
15: 721–73730185915
10.1038/s41575-018-0054-1PMC7097092MukherjeeT
 (2019) NOD1 and NOD2 in inflammation, immunity and disease. Arch Biochem Biophys
670: 69–8130578751
10.1016/j.abb.2018.12.022NielsenAE
 (2017) Synthetic agonists of NOD-like, RIG-I-like, and C-type lectin receptors for probing the inflammatory immune response. Future Med Chem
9: 1345–136028776416
10.4155/fmc-2017-0101

## RIG-I-like receptor family

Catalytic receptors → Pattern recognition receptors → RIG-I-like receptor family

### Overview:

There are three human RIG-I-like receptors (RLRs) which are cytoplasmic pattern recognition receptors (PRRs) of the innate immune system. They detect non-self cytosolic double-stranded RNA species and and 5’-triphosphate single-stranded RNA from various sources and are essential for inducing production of type I interferons, such as IFNβ, type III interferons, and other anti-pathogenic effectors [27, 28]. They function as RNA helicases (EC 3.6.4.13) using the energy from ATP hydrolysis to unwind RNA.

**Table:** 

Nomenclature	DExD/H-box helicase 58	interferon induced with helicase C domain 1	DExH-box helicase 58
Common abbreviation	RIG-1	MDA5	LGP2
HGNC, UniProt	*RIGI*, O95786	*IFIH1*, Q9BYX4	*DHX58*, Q96C10
EC number	3.6.4.13	3.6.4.13	3.6.4.13
	RNA helicase: uses the energy from ATP hydrolysis to unwind RNA: ATP + H(2)O <=> ADP + phosphate	RNA helicase; uses the energy from ATP to unwind RNA: ATP + H(2)O <=> ADP + phosphate	RNA helicase: uses the energy from ATP hydrolysis to unwind RNA: ATP + H(2)O <=> ADP + phosphate

Further reading on RIG-I-like receptor familyChowKT
 (2018) RIG-I and Other RNA Sensors in Antiviral Immunity. Annu Rev Immunol
36: 667–69429677479
10.1146/annurev-immunol-042617-053309KatoH
 (2015) RIG-I-like receptors and autoimmune diseases. Curr Opin Immunol
37: 40–526530735
10.1016/j.coi.2015.10.002LässigC
 (2017) Discrimination of cytosolic self and non-self RNA by RIG-I-like receptors. J Biol Chem
292: 9000–900928411239
10.1074/jbc.R117.788398PMC5454087MaZ
 (2018) Innate Sensing of DNA Virus Genomes. Annu Rev Virol
5: 341–36230265633
10.1146/annurev-virology-092917-043244PMC6443256RehwinkelJ
 (2020) RIG-I-like receptors: their regulation and roles in RNA sensing. Nat Rev Immunol
20: 537–55132203325
10.1038/s41577-020-0288-3PMC7094958YongHY
 (2018) RIG-I-Like Receptors as Novel Targets for Pan-Antivirals and Vaccine Adjuvants Against Emerging and Re-Emerging Viral Infections. Front Immunol
9: 137929973930
10.3389/fimmu.2018.01379PMC6019452

Further reading on Pattern recognition receptorsBrozP
 (2016) Inflammasomes: mechanism of assembly, regulation and signalling. Nat Rev Immunol
16: 407–2027291964
10.1038/nri.2016.58BryantCE
 (2015) Advances in Toll-like receptor biology: Modes of activation by diverse stimuli. Crit Rev Biochem Mol Biol
50: 359–7925857820
10.3109/10409238.2015.1033511FeerickCL
 (2017) Understanding the regulation of pattern recognition receptors in inflammatory diseases - a ‘Nod’ in the right direction. Immunology
150: 237–24727706808
10.1111/imm.12677PMC5290251RathinamVA
 (2016) Inflammasome Complexes: Emerging Mechanisms and Effector Functions. Cell
165: 792–80027153493
10.1016/j.cell.2016.03.046PMC5503689UnterholznerL. (2013) The interferon response to intracellular DNA: why so many receptors?
Immunobiology
218: 1312–2123962476
10.1016/j.imbio.2013.07.007YinQ
 (2015) Structural biology of innate immunity. Annu Rev Immunol
33: 393–41625622194
10.1146/annurev-immunol-032414-112258PMC4394028

## Receptor guanylyl cyclase (RGC) family

Catalytic receptors → Receptor guanylyl cyclase (RGC) family

### Overview:

The mammalian genome encodes seven guanylyl cyclases, GC-A to GC-G, that are homodimeric transmembrane receptors activated by a diverse range of endogenous ligands. These enzymes convert guanosine-5’-triphosphate to the intracellular second messenger cyclic guanosine-3’,5’-monophosphate (cyclic GMP). GC-A, GC-B and GC-C are expressed predominantly in the cardiovascular system, skeletal system and intestinal epithelium, respectively. GC-D and GC-G are found in the olfactory neuropepithelium and Grueneberg ganglion of rodents, respectively. GC-E and GC-F are expressed in retinal photoreceptors.

## Transmembrane guanylyl cyclases

Catalytic receptors → Receptor guanylyl cyclase (RGC) family → Transmembrane guanylyl cyclases

### Overview:

Transmembrane guanylyl cyclases are homodimeric receptors activated by a diverse range of endogenous ligands. GC-A, GC-B and GC-C are expressed predominantly in the cardiovascular system, skeletal system and intestinal epithelium, respectively. GC-D and GC-G are found in the olfactory neuropepithelium and Grüeneberg ganglion of rodents, respectively. GC-E and GC-F are expressed in retinal photoreceptors. Family members have conserved ligand-binding, catalytic (guanylyl cyclase) and regulatory domains with the exception of NPR-C which has an extracellular binding domain homologous to that of other NPRs, but with a truncated intracellular domain which appears to couple, *via* the G_i/o_ family of G proteins, to activation of phospholipase C, inwardly-rectifying potassium channels and inhibition of adenylyl cyclase activity [198].

**Table:** 

Nomenclature	Guanylyl cyclase-A	Guanylyl cyclase-B	Guanylyl cyclase-C	natriuretic peptide receptor 3
Common abbreviation	GC-A	GC-B	GC-C	NPR-C
HGNC, UniProt	*NPR1*, P16066	*NPR2*, P20594	*GUCY2C*, P25092	*NPR3*, P17342
Potency order	atrial natriuretic peptide (*NPPA*, P01160) ≥ brain natriuretic peptide (*NPPB*, P16860) » C-type natriuretic peptide (*NPPC*, P23582) [257]	C-type natriuretic peptide (*NPPC*, P23582) » atrial natriuretic peptide (*NPPA*, P01160) » brain natriuretic peptide (*NPPB*, P16860) [257]	uroguanylin (*GUCA2B*, Q16661) > guanylin (*GUCA2A*, Q02747)	atrial natriuretic peptide (*NPPA*, P01160) > C-type natriuretic peptide (*NPPC*, P23582) ≥ brain natriuretic peptide (*NPPB*, P16860) [257]
Endogenous agonists	atrial natriuretic peptide (*NPPA*, P01160) (Binding) [211], brain natriuretic peptide (*NPPB*, P16860) (Binding) [211], mutant ANP [37, 187]	C-type natriuretic peptide (*NPPC*, P23582) (Binding) [257]	guanylin (*GUCA2A*, Q02747) (Binding), uroguanylin (*GUCA2B*, Q16661) (Binding)	–
Agonists	–	–	dolcanatide [241]	–
Selective agonists	*Dendroaspis* natriuretic peptide [246], PL-3994 [61], compound 20 [5], cenderitide [181], sANP [211]	MCUF-42 [173], cenderitide [181], vosoritide [171]	linaclotide [30, 103], *E. coli* heat-stable enterotoxin (ST_a_) [30], plecanatide [240]	cANF^4-23^ [174]
Endogenous antagonists	–	–	–	osteocrin (*OSTN*, P61366) [192, 203]
Selective antagonists	A-71915 (p*K*_i_ 9.2–9.5) [54], [Asu7,23’] β-ANP-(7-28) (p*K*_i_ 7.5) [133], HS-142-1 [194], anantin [280, 292]	peptide P19 (p*K*_d_ 7.8) [56], HS-142-1 [194], [Ser^11^](N-CNP,C-ANP)pBNP^2-15^ [56], compound C10 ^[10]^	–	M372049 [116]
Labelled ligands	[^125^I]ANP (human) (Agonist)	[^125^I]CNP (human)	[^125^I]St_a_ (Agonist) [101]	[^125^I]ANP (human)

**Table:** 

Nomenclature	Guanylyl cyclase-D	Guanylyl cyclase-E	Guanylyl cyclase-F	Guanylyl cyclase-G
Common abbreviation	GC-D	GC-E	GC-F	GC-G
HGNC, UniProt	–	*GUCY2D*, Q02846	*GUCY2F*, P51841	*GUCY2GP*, –
Localisation	–	Retinal photoreceptors	Retinal photoreceptors	Grüneberg ganglion
Principal function(s)	–	Vision/phototransduction	Vision/phototransduction	Thermosensation
Endogenous ligands	–	–	–	Cold
Comments	Pseudogene in humans	–	–	Pseudogene in humans

### Comments:

GC-D and GC-G have been reported to be activated intracellularly by guanylyl cyclase-activating protein 1 (*GUCA1A*, P43080) and guanylyl cyclase-activating protein 2 (*GUCA1B*, Q9UMX6). GC-D and GC-G may be activated by atmospheric levels of CO_2_ through the formation of intracellular bicarbonate ions [36, 117]. GC-G may be activated at cooler temperatures (20-25°C) through apparent stabilisation of the dimer [35].

## Nitric oxide (NO)-sensitive (soluble) guanylyl cyclase

Catalytic receptors → Receptor guanylyl cyclase (RGC) family → Nitric oxide (NO)-sensitive (soluble) guanylyl cyclase

### Overview:

Nitric oxide (NO)-sensitive (soluble) guanylyl cyclase (GTP diphosphate-lyase (cyclising)), E.C. 4.6.1.2, is a heterodimer comprising a β_1_ subunit and one of two alpha subunits (α_1_, α_2_) giving rise to two functional isoforms, GC-1 (α_1_β_1_) and GC-2 (α_2_β_1_) [229, 301]. A haem group is associated with the β subunit and is the target for the endogenous ligand NO, and, potentially, carbon monoxide [77].

### Complexes

**Table:** 

Nomenclature	Guanylyl cyclase, α_1_β_2_	Guanylyl cyclase, α_2_β_1_
Common abbreviation	GC-1	GC-2
Subunits	Guanylyl cyclase α_1_ subunit, Guanylyl cyclase β_1_ subunit	Guanylyl cyclase α_2_ subunit, Guanylyl cyclase β_1_ subunit
EC number	4.6.1.2	4.6.1.2
Endogenous ligands	NO	NO
Selective activators	praliciguat (pEC_50_ 6.6) [263], YC-1 [77, 143, 229], ataciguat [235], cinaciguat [apo-GC-1] [251], olinoiguat [31], riociguat [249, 250], runcaciguat [100], vericiguat [70]	YC-1 [143, 229], cinaciguat [apo-GC-2] [251], olinciguat [31], riociguat [249, 250]
Selective inhibitors	NS 2028 (pIC_50_ 8.1) [209] – Bovine, ODQ (pIC_50_ 7.5) [84]	ODQ

### Subunits

**Table:** 

Nomenclature	Guanylyl cyclase α_1_ subunit	Guanylyl cyclase α_2_ subunit	Guanylyl cyclase β_1_ subunit	Guanylyl cyclase β_2_ subunit
HGNC, UniProt	*GUCY1A1*, Q02108	*GUCY1A2*, P33402	*GUCY1B1*, Q02153	*GUCY1B2*, O75343

### Comments:

ODQ also shows activity at other haem-containing proteins [68], while YC-1 may also inhibit cGMP-hydrolysing phosphodiesterases [76, 80].

Further reading on Receptor guanylyl cyclase (RGC) familyKuhnM. (2016) Molecular Physiology of Membrane Guanylyl Cyclase Receptors. Physiol Rev
96: 751–80427030537
10.1152/physrev.00022.2015WaldmanSA
 (2018) Guanylate cyclase-C as a therapeutic target in gastrointestinal disorders. Gut
67: 1543–155229563144
10.1136/gutjnl-2018-316029PMC6204952

## Receptor tyrosine kinases (RTKs)

Catalytic receptors → Receptor kinases → TK: Tyrosine kinase → Receptor tyrosine kinases (RTKs)

### Overview:

Receptor tyrosine kinases (RTKs), a family of cell-surface receptors, which transduce signals to polypeptide and protein hormones, cytokines and growth factors are key regulators of critical cellular processes, such as proliferation and differentiation, cell survival and metabolism, cell migration and cell cycle control [20, 93, 271]. In the human genome, 58 RTKs have been identified, which fall into 20 families [156].

All RTKs display an extracellular ligand binding domain, a single transmembrane helix, a cytoplasmic region containing the protein tyrosine kinase activity (occasionally split into two domains by an insertion, termed the kinase insertion), with juxta-membrane and C-terminal regulatory regions. Agonist binding to the extracellular domain evokes dimerization, and sometimes oligomerization, of RTKs (a small subset of RTKs forms multimers even in the absence of activating ligand). This leads to autophosphorylation in the tyrosine kinase domain in a trans orientation, serving as a site of assembly of protein complexes and stimulation of multiple signal transduction pathways, including phospholipase C-γ, mitogen-activated protein kinases and phosphatidylinositol 3-kinase [271].

RTKs are of widespread interest not only through physiological functions, but also as drug targets in many types of cancer and other disease states. Many diseases result from genetic changes or abnormalities that either alter the activity, abundance, cellular distribution and/or regulation of RTKs. Therefore, drugs that modify the dysregulated functions of these RTKs have been developed which fall into two categories. One group is often described as ‘biologicals’, which block the activation of RTKs directly or by chelating the cognate ligands, while the second are small molecules designed to inhibit the tyrosine kinase activity directly.

## Type I RTKs: ErbB (epidermal growth factor) receptor family

Catalytic receptors → Receptor kinases → TK: Tyrosine kinase → Receptor tyrosine kinases (RTKs) → Type I RTKs: ErbB (epidermal growth factor) receptor family

### Overview:

ErbB family receptors are Class I receptor tyrosine kinases [93]. EGFR (HER1) was the first member of the family to be identified. ERBB2 (also known as HER-2 or NEU) appears to act as an essential partner for the other members of the family without itself being activated by a cognate ligand [94]. Ligands of the ErbB family of receptors are peptides, many of which are generated by proteolytic cleavage of cell-surface proteins. HER/ErbB is the viral counterpart to the receptor tyrosine kinase EGFR. All family members heterodimerize with each other to activate downstream signalling pathways and are aberrantly expressed in many cancers, particularly forms of breast cancer and lung cancer. Mutations in the EGFR are responsible for acquired resistance to tyrosine kinase inhibitor chemotherapeutics.

**Table:** 

Nomenclature	epidermal growth factor receptor	erb-b2 receptor tyrosine kinase 2	erb-b2 receptor tyrosine kinase 3	erb-b2 receptor tyrosine kinase 4
Common abbreviation	EGFR	HER2	HER3	HER4
HGNC, UniProt	*EGFR*, P00533	*ERBB2*, P04626	*ERBB3*, P21860	*ERBB4*, Q15303
EC number	2.7.10.1	2.7.10.1	2.7.10.1	2.7.10.1
Endogenous ligands	EGF (*EGF*, P01133) (Binding) (p*K*_i_ 8.9) [44], betacellulin (*BTC*, P35070) (Binding) (p*K*_i_ 8.4) [44], HB-EGF (*HBEGF*, Q99075) (Binding) (p*K*_i_ 8.4) [44], TGFα (*TGFA*, P01135) (Binding) (p*K_i_* 6.8) [44], amphiregulin (*AREG*, P155114) (Binding), epigen (*EPGN*, Q6UW88) (Binding), epiregulin (*EREG*, O14944) (Binding)	–	neuregulin-1 (NRG1, Q02297) (NRG-1α) (pIC_50_ 9.3) [131], neuregulin-1 *(NRG1*, Q02297) (NRG-1β) (p*K*_d_ 8.9) [269]	neuregulin-1 (NRG1, Q02297) (NRG-1β) (p*K*_d_ 9.1) [269], betacellulin (BTC, P35070) (BTCβ) (pIC_50_ 8.4) [131], neuregulin-1 (NRG1, Q02297) (NRG-1α) (pIC_50_ 6.3) [131]
Ligands	–	–	neuregulin-1 (*NRG1*, Q02297) (NRG-1β) (pIC_50_ 8.3) [131]	neuregulin-1 (*NRG1*, Q02297) (NRG-1β) (pIC_50_ 8.3) [131]
Inhibitors	afatinib (p*K*_d_ 9.6) [51], tesevatinib (pIC_50_ 9.5) [86], afatinib (ex19del EGFR) (pIC_50_ 9.2) [47], erlotinib (ex19del EGFR) (pIC_50_ 8.2) [47], osimertinib (ex19del/T790 M EGFR) (pIC_50_ 8.2) [47], gefitinib (ex19del EGFR) (pIC_50_ 8.1) [47], lapatinib (pIC_50_ 8) [228], afatinib (WT EGFR) (pIC_50_ 7.8) [47], gefitinib (WT EGFR) (pIC_50_ 7.2) [47], erlotinib (WT EGFR) (pIC_50_ 7) [47], osimertinib (WT EGFR) (pIC_50_ 6.3) [47]	lapatinib (p*K*_i_ 7.9) [290]	–	poziotinib (pIC_50_ 7.6) [201]
Antibodies	panitumumab (Antagonist) (p*K*_d_ 10.3) [72], necitumumab (Binding) (p*K_d_* 9.5) [168], cetuximab (Binding) (p*K*_d_ 9.4) [89]	pertuzumab (Inhibition) (pIC_50_>8) [134], trastuzumab (Inhibition)	–	–

### Comments:

[^125^I]EGF (human) has been used to label the ErbB1 EGF receptor. The extracellular domain of ErbB2 can be targetted by the antibodies trastuzumab and pertuzumab to inhibit ErbB family action. The intracellular ATP-binding site of the tyrosine kinase domain can be inhibited by GW583340 (7.9-8.0, [85]), gefitinib, erlotinib and tyrphostins AG879 and AG1478.

Further reading on Type I RTKs: ErbB (epidermal growth factor) receptor familyKobayashiY
 (2016) Not all epidermal growth factor receptor mutations in lung cancer are created equal: Perspectives for individualized treatment strategy. Cancer Sci
107: 1179–8627323238
10.1111/cas.12996PMC5021039

## Type II RTKs: Insulin receptor family

Catalytic receptors → Receptor kinases → TK: Tyrosine kinase → Receptor tyrosine kinases (RTKs) → Type II RTKs: Insulin receptor family

### Overview:

The circulating peptide hormones insulin (*INS*, P01308) and the related insulin-like growth factors (IGF) activate Class II receptor tyrosine kinases [93], to evoke cellular responses, mediated through multiple intracellular adaptor proteins. Unlike other receptor tyrosine kinases, the functional receptor in the insulin receptor family is derived from a single gene product, cleaved post-translationally into two peptides, which then cross-link via disulphide bridges to form a heterotetramer. Intriguingly, the endogenous peptide ligands are formed in a parallel fashion with post-translational processing producing a heterodimer linked by disulphide bridges. Signalling through the receptors is mediated through a rapid autophosphorylation event at intracellular tyrosine residues, followed by recruitment of multiple adaptor proteins, notably *IRS1* (P35568), *IRS2* (Q9Y4H2), *SHC1* (P29353), *GRB2* (P62993) and *SOS1* (Q07889).

Serum levels of free IGFs are kept low by the action of IGF binding proteins (IGFBP1-5, P08833, P18065, P17936, P22692, P24593), which sequester the IGFs; overexpression of IGFBPs may induce apoptosis, while IGFBP levels are also altered in some cancers.

**Table:** 

Nomenclature	Insulin receptor	Insulin-like growth factor I receptor	Insulin receptor-related receptor
Common abbreviation	InsR	IGF1R	IRR
HGNC, UniProt	*INSR*, P06213	*IGF1R*, P08069	*INSRR*, P14616
EC number	2.7.10.1	2.7.10.1	2.7.10.1
Endogenous agonist rank potency	insulin (*INS*, P01308) > insulin-like growth factor 1 (*IGF1*, P05019)	–	–
Inhibitors	–	BMS-754807 (pIC_50_ 8.7) [287], GSK-1838705A (pIC_50_ 8.7) [231], GSK-1838705A *(p*K*_d_* 8.1) [51], PQ401 (pIC_50_>6) [79], AG 1024 (pIC_50_ 4.7) [215]	–
Selective inhibitors	–	NVP-AEW541 (pIC_50_ 9.4) [83]	-
Endogenous agonists	insulin (*INS*, P01308), insulin-like growth factor 1 (*IGF1*, P05019)	insulin-like growth factor 1 (*IGF1*, P05019), insulin-like growth factor 2 (*IGF2*, P01344)	–

### Comments:

There is evidence for low potency binding and activation of insulin receptors by IGF1. IGF2 also binds and activates the cation-independent mannose 6-phosphate receptor (also known as the insulin-like growth factor 2 receptor; *IGF2R*; P11717), which lacks classical signalling capacity and appears to subserve a trafficking role [175]. INSRR, which has a much more discrete localization, being predominant in the kidney [146], currently lacks a cognate ligand or evidence for functional impact.

Antibodies targetting IGF1, IGF2 and the extracellular portion of the IGF1 receptor are in clinical trials. PQ401 inhibits the insulin-like growth factor receptor [5], while BMS-536924 inhibits both the insulin receptor and the insulin-like growth factor receptor [286].

## Type III RTKs: PDGFR, CSFR, Kit, FLT3 receptor family

Catalytic receptors → Receptor kinases → TK: Tyrosine kinase → Receptor tyrosine kinases (RTKs) → Type III RTKs: PDGFR, CSFR, Kit, FLT3 receptor family

### Overview:

Type III RTKs include PDGFR, CSF-1R (Ems), Kit and FLT3, which function as homo- or heterodimers. Endogenous ligands of PDGF receptors are homo- or heterodimeric: PDGFA, PDGFB, VEGFE and PDGFD (*PDGFD*, Q9GZP0) combine as homo- or heterodimers to activate homo- or heterodimeric PDGF receptors. SCF is a dimeric ligand for KIT. Ligands for CSF1R are either monomeric or dimeric glycoproteins, while the endogenous agonist for FLT3 is a homodimer.

**Table:** 

Nomenclature	platelet derived growth factor receptor alpha	platelet derived growth factor receptor beta	KIT proto-oncogene, receptor tyrosine kinase
Common abbreviation	PDGFRα	PDGFRβ	Kit
HGNC, UniProt	*PDGFRA*, P16234	PDGFRB, P09619	*KIT*, P10721
EC number	2.7.10.1	2.7.10.1	2.7.10.1
Endogenous ligands	PDGF	PDGF	–
Inhibitors	PP121 (pIC_50_ 8.7) [7], crenolanib (p*K*_d_ 8.7) [108], EN-MD-2076 (pIC_50_ 7.2) [219]	crenolanib (p*K*_d_ 8.5) [108], SU-14813 (pIC_50_ 8.4) [216], famitinib (pIC_50_ 8.4) [38], sunitinib (pIC_50_ 8.2) [141], sunitinib (p*K*_i_ 8.1) [189]	sunitinib (p*K*_d_ 9.4) [51], famitinib (pIC_50_ 8.7) [38], masitinib (p*K*_d_ 8.1) [51], SU-14813 (pIC_50_ 7.8) [216], AKN-028 (pIC_50_ 7.5) [65], sorafenib (pIC_50_ 7.2) [285]
Selective inhibitors	CP-673451 (pIC_50_ 8) [226]	CP-673451 (pIC_50_ 9) [226]	–
Endogenous agonists	–	–	stem cell factor (KITLG, P21583) [268]

**Table:** 

Nomenclature	colony stimulating factor 1 receptor	fms related receptor tyrosine kinase 3
Common abbreviation	CSFR	FLT3
HGNC, UniProt	*CSF1R*, P07333	*FLT3*, P36888
EC number	2.7.10.1	2.7.10.1
Endogenous ligands	G-CSF (*CSF3*, P09919), IL-34 (*IL34*, Q6ZMJ4), M-CSF (*CSF1*, P09603)	Fms-related tyrosine kinase 3 ligand (*FLT3LG*, P49771)
Inhibitors	JNJ-28312141 (pIC_50_ 9.2) [177], Ki-20227 (p*K*_d_ 9.1) [51], Ki-20227 (pIC_50_ 8.7) [208], GW-2580 (p*K*_d_ 8.7) [51], JNJ-28312141 (p*K*_d_ 8.5) [51]	AC710 (p*K*_d_ 9.3) [165], linifanib (p*K*_d_ 9.2) [51], crenolanib (p*K*_d_ 9.1) [108], EN-MD-2076 (pIC_50_ 8.5) [219], tandutinib (p*K*_d_ 8.5) [51], tandutinib (pIC_50_ 6.7) [136]
Selective inhibitors	GW-2580 (pIC_50_ 7.2) [46]	denfivontinib (pIC_50_ 9.4) [153]
Comments	Upregulation of CSF1R expression is associated with migroglial activation and immune pathology in Alzhermer’s disease (AD) [90, 91]. Pharmacological inhibition of CSF1R with GW-2580 reduces microglial proliferation and prevents disease progression in a mouse model of AD, but this does not correlate with amyloid-p plaque numbers [210].	5’-fluoroindirubinoxime has been described as a selective FLT3 inhibitor [39].

### Comments:

Various small molecular inhibitors of type III RTKs have been described, including imatinib and nilotinib (targetting PDGFR, KIT and CSF1R); midostaurin and AC220 (quizartinib; FLT3), as well as pan-type III RTK inhibitors such as sunitinib and sorafenib [217]; 5’-fluoroindirubinoxime has been described as a selective FLT3 inhibitor [2].

## Type IV RTKs: VEGF (vascular endothelial growth factor) receptor family

Catalytic receptors → Receptor kinases → TK: Tyrosine kinase → Receptor tyrosine kinases (RTKs) → Type IV RTKs: VEGF (vascular endothelial growth factor) receptor family

### Overview:

VEGF receptors are homo- and heterodimeric proteins, which are characterized by seven Ig-like loops in their extracellular domains and a split kinase domain in the cytoplasmic region. They are key regulators of angiogenesis and lymphangiogenesis; as such, they have been the focus of drug discovery for conditions such as metastatic cancer. Splice variants of VEGFR1 and VEGFR2 generate truncated proteins limited to the extracellular domains, capable of homodimerisation and binding VEGF ligands as a soluble, non-signalling entity. Ligands at VEGF receptors are typically homodimeric. VEGFA (*VEGFA*, P15692) is able to activate VEGFR1 and VEGFR2 homodimers, VEGFR1/2 heterodimers and VEGFR2/3 heterodimers. VEGFB (*VEGFB*, P49765) and placental growth factor (*PGF*, P49763) activate VEGFR1 homodimers, while VEGFC (*VEGFC*, P49767) and VEGFD (*VEGFD*, O43915) activate VEGFR2/3 heterodimers and VEGFR3 homodimers, and, following proteolysis, VEGFR2 homodimers.

**Table:** 

Nomenclature	fms related receptor tyrosine kinase 1	kinase insert domain receptor	fms related receptor tyrosine kinase 4
Common abbreviation	VEGFR-1	VEGFR-2	VEGFR-3
HGNC, UniProt	*FLT1*, P17948	*KDR*, P35968	*FLT4*, P35916
EC number	2.7.10.1	2.7.10.1	2.7.10.1
Endogenous ligands	VEGFA (*VEGFA*, P15692), VEGFB (*VEGFB*, P49765)	VEGFA (*VEGFA*, P15692), VEGFC (*VEGFC*, P49767), VEGFE (*PDGFC*, Q9NRA1)	VEGFC (*VEGFC*, P49767), VEGFD (*VEGFD*, O43915), VEGFE (*PDGFC*, Q9NRA1)
Inhibitors	SU-14813 (pIC_50_ 8.7) [216], CEP-11981 (pIC_50_ 8.5) [119], semaxanib (pIC_50_ 8.1) [21]	cabozantinib (pIC_50_ 10.5) [293], axitinib (pIC_50_ 9.6) [154], foretinib (pIC_50_ 8.2–9.1) [199], cediranib (p*K*_d_ 9) [51], tesevatinib (pIC_50_ 8.8) [86], motesanib (p*K*_d_ 8.6) [51], famitinib (pIC_50_ 8.3) [38], axitinib (p*K*_d_ 8.2) [51]	tesevatinib (pIC_50_ 8.1) [86], sunitinib (pIC_50_ 8.1) [137], nintedanib (pIC_50_ 7.9) [114]
Sub/family-selective inhibitors	pazopanib (pIC_50_ 8) [104]	pazopanib (p*K*_d_ 7.8) [51], pazopanib (pIC_50_ 7.5) [104]	pazopanib (pIC_50_ 7.3) [104]
Antibodies	–	ramucirumab (Antagonist) (pIC_50_ 9) [172]	–

### Comments:

The VEGFR, as well as VEGF ligands, have been targeted by antibodies and tyrosine kinase inhibitors. DMH4 [74], Ki8751 [145] and ZM323881, a novel inhibitor of vascular endothelial growth factor-receptor-2 tyrosine kinase activity [283] are described as VEGFR2-selective tyrosine kinase inhibitors. Bevacizumab is a monoclonal antibody directed against VEGF-A, used clinically for the treatment of certain metastatic cancers; an antibody fragment has been used for wet age-related macular degeneration.

## Type V RTKs: FGF (fibroblast growth factor) receptor family

Catalytic receptors → Receptor kinases → TK: Tyrosine kinase → Receptor tyrosine kinases (RTKs) → Type V RTKs: FGF (fibroblast growth factor) receptor family

### Overview:

Fibroblast growth factor (FGF) family receptors act as homo- and heterodimers, and are characterized by Ig-like loops in the extracellular domain, in which disulphide bridges may form across protein partners to allow the formation of covalent dimers which may be constitutively active. FGF receptors have been implicated in achondroplasia, angiogenesis and numerous congenital disorders. At least 22 members of the FGF gene family have been identified in the human genome [11]. Within this group, subfamilies of FGF may be divided into canonical, intracellular and hormone-like FGFs. FGF1-FGF10 have been identified to act through FGF receptors, while FGF11-14 appear to signal through intracellular targets. Other family members are less well characterized [282].

Approved FGFR inhibitors include erdafitinib, pemigatinib, and futibatinib. Common adverse effects associated with these drugs, and approaches for their management are reviewed in [256].

**Table:** 

Nomenclature	fibroblast growth factor receptor 1	fibroblast growth factor receptor 2	fibroblast growth factor receptor 3	fibroblast growth factor receptor 4
Common abbreviation	FGFR1	FGFR2	FGFR3	FGFR4
HGNC, UniProt	*FGFR1*, P11362	*FGFR2*, P21802	*FGFR3*, P22607	*FGFR4*, P22455
EC number	2.7.10.1	2.7.10.1	2.7.10.1	2.7.10.1
Endogenous ligands	FGF-1 (*FGF1*, P05230), FGF-2 (*FGF2*, P09038), FGF-4 (*FGF4*, P08620) > FGF-5 (*FGF5*, P12034), FGF-6 (*FGF6*, P10767) [213]	FGF-10 (*FGF10*, O15520) [298] FGF-1 (*FGF1*, P05230) > FGF-4 (*FGF4*, P08620), FGF-7 (*FGF7*, P21781), FGF-9 (*FGF9*, P31371) > FGF-2 (*FGF2*, P09038), FGF-6 (*FGF6*, P10767) [213]	FGF-3 (*FGF3*, P11487) FGF-1 (*FGF1*, P05230), FGF-2 (*FGF2*, P09038), FGF-9 (*FGF9*, P31371) > FGF-4 (*FGF4*, P08620), FGF-8 (*FGF8*, P55075) [213]	FGF-1 (*FGF1*, P05230), FGF-2 (*FGF2*, P09038), FGF-4 (*FGF4*, P08620), FGF-9 (*FGF9*, P31371) > FGF-6 (*FGF6*, P10767), FGF-8 (*FGF8*, P55075): FGF-19 (*FGF19*, 095750) [213]
Sub/family-selective inhibitors	LY2874455 (pIC_50_ 8.6) [303]	LY2874455 (pIC_50_ 8.6) [303]	LY2874455 (pIC_50_ 8.2) [303]	LY2874455 (pIC_50_ 8.2) [303]
Selective inhibitors	–	–	–	BLU-9931 (Irreversible inhibition) (pIC_50_ 8.5) [99]
Agonists	–	palifermin	–	–

### Comments:

Splice variation of the receptors can influence agonist responses. *FGFRL1* (Q8N441) is a truncated kinase-null analogue.

Various antibodies and tyrosine kinase inhibitors have been developed against FGF receptors [160, 307]. PD161570 is an FGFR tyrosine kinase inhibitor [13], while PD173074 has been described to inhibit FGFR1 and FGFR3 [247].

Further reading on Type V RTKs: FGF (fibroblast growth factor) receptor familySubbiahV
 (2023) Clinical development and management of adverse events associated with FGFR inhibitors. Cell Rep Med
4: 10120437757826
10.1016/j.xcrm.2023.101204PMC10591034

## Type VI RTKs: PTK7/CCK4

Catalytic receptors → Receptor kinases → TK: Tyrosine kinase → Receptor tyrosine kinases (RTKs) → Type VI RTKs: PTK7/CCK4

### Overview:

The PTK7 receptor is associated with polarization of epithelial cells and the development of neural structures. Sequence analysis suggests that the gene product is catalytically inactive as a protein kinase, hence acting as a pseudokinase. There is, however, evidence for a role in Wnt signalling [22,2], as well as an ability to form heteromers with other RTKs, such as VEGFR2 and ROR2.

**Table:** 

Nomenclature	protein tyrosine kinase 7 (inactive)
Common abbreviation	CCK4
HGNC, UniProt	*PTK7*, Q13308
EC number	2.7.10.1

Further reading on Type VI RTKs: PTK7/CCK4DessauxC
 (2024) Recent insights into the therapeutic strategies targeting the pseudokinase PTK7 in cancer. Oncogene
43: 1973–198438773263
10.1038/s41388-024-03060-xPMC11196218

## Type VII RTKs: Neurotrophin receptor/Trk family

Catalytic receptors → Receptor kinases → TK: Tyrosine kinase → Receptor tyrosine kinases (RTKs) → Type VII RTKs: Neurotrophin receptor/Trk family

### Overview:

The neurotrophin receptor family of RTKs include tropomyosin-related kinase (Trk) receptors TrkA, TrkB and TrkC. Their cognate ligands are NGF, BDNF and neurotrophin-3, respectively. They are associated primarily with proliferative and migration effects in neural systems. Various isoforms of neurotrophin receptors exist, including truncated forms of TrkB and TrkC, which lack catalytic domains. p75 (TNFRSF16, also known as nerve growth factor receptor) can interact with neurotrophins and their propeptides. While p75 can lead to apoptosis, it can also form a complex with Trk receptors to mediate survival signalling [45].

**Table:** 

Nomenclature	neurotrophic receptor tyrosine kinase 1	neurotrophic receptor tyrosine kinase 2	neurotrophic receptor tyrosine kinase 3
Common abbreviation	TrkA	TrkB	TrkC
HGNC, UniProt	*NTRK1*, P04629	*NTRK2*, Q16620	*NTRK3*, Q16288
EC number	2.7.10.1	2.7.10.1	2.7.10.1
Endogenous ligands	NGF (*NGF*, P01138) > neurotrophin-3 (*NTF3*, P20783)	BDNF (*BDNF*, P23560), neurotrophin-4 (*NTF4*, P34130) > neurotrophin-3 (*NTF3*, P20783)	neurotrophin-3 (*NTF3*, P20783)
Inhibitors	selitrectinib (pIC_50_>9.3) [202], compound 2c (pIC_50_ 8.9) [277], milciclib (pIC_50_ 7.3) [23], ONO-7579 [125]	ONO-7579 [125]	ONO-7579 [125]
Sub/family-selective inhibitors	AZD1332 (pIC_50_>8.3) [9], GNF-5837 (pIC_50_ 8) [2]	AZD1332 (pIC_50_>8.3) [9], GNF-5837 (pIC_50_ 8.1) [2]	AZD1332 (pIC_50_>8.3) [9], GNF-5837 (pIC_50_ 8.1) [2]

### Comments:

[^125^I]NGF (human) and [^125^I]BDNF (human) have been used to label the TrkA and TrkB receptor, respectively. p75 influences the binding of NGF (*NGF*, P01138) and neurotroph-in-3 (*NTF3*, P20783) to TrkA. The ligand selectivity of p75 appears to be dependent on the cell type; for example, in sympathetic neurons, it binds neurotrophin-3 (*NTF3*, P20783) with comparable affinity to TrkC [53].

Small molecule agonists of trkB have been described, including LM22A4 [185], while ANA12 has been described as a non-competitive antagonist of BDNF binding to trkB [34]. GNF5837 is a family-selective tyrosine kinase inhibitor [2], while the tyrosine kinase activity of the TrkA receptor can be inhibited by GW441756 (pIC_50_= 8.7, [289]) and tyrphostin AG879 [207].

## Type VIII RTKs: ROR family

Catalytic receptors → Receptor kinases → TK: Tyrosine kinase → Receptor tyrosine kinases (RTKs) → Type VIII RTKs: ROR family

### Overview:

ROR1 and ROR2 are involved in regulating Wnt-5a (*WNT5A*, P41221) signalling. There is evidence that ROR1 and ROR2 can form heteromeric complexes. Due to their role in cancer, therapies targeting RORs are under investigation. Thus, ROR1 and ROR2 appear to be activated by Wnt-5a binding to a Frizzled receptor thereby forming a cell-surface multiprotein complex [96].

**Table:** 

Nomenclature	receptor tyrosine kinase like orphan receptor 1	receptor tyrosine kinase like orphan receptor 2
Common abbreviation	ROR1	ROR2
HGNC, UniProt	*ROR1*, Q01973	*ROR2*, Q01974
EC number	2.7.10.1	2.7.10.1

Further reading on Type VIII RTKs: ROR familyEndoM
 (2022) The Ror-Family Receptors in Development, Tissue Regeneration and Age-Related Disease. Front Cell Dev Biol
10: 89176335493090
10.3389/fcell.2022.891763PMC9043558MenckK
 (2021) The WNT/ROR Pathway in Cancer: From Signaling to Therapeutic Intervention. Cells
1010.3390/cells10010142PMC782817233445713TiguAB
 (2024) Therapeutic advances in the targeting of ROR1 in hematological cancers. Cell Death Discov
10: 47139551787
10.1038/s41420-024-02239-1PMC11570672

## Type IX RTKs: MuSK

Catalytic receptors → Receptor kinases → TK: Tyrosine kinase → Receptor tyrosine kinases (RTKs) → Type IX RTKs: MuSK

### Overview:

The muscle-specific kinase MuSK is associated with the formation and organisation of the neuromuscular junction from the skeletal muscle side. Agrin (*AGRN*, O00468) forms a complex with low-density lipoprotein receptor-related protein 4 (*LRP4*, O75096) to activate MuSK [139]. MuSK-mediated phosphorylation of downstream targets is involved in stabilised neuromuscular function. It is the target of pathogenic autoantibodies in myasthenia gravis, an autoimmune neuromuscular disease.

**Table:** 

Nomenclature	muscle associated receptor tyrosine kinase
Common abbreviation	MuSK
HGNC, UniProt	*MUSK*, O15146
EC number	2.7.10.1

### Comments:

Thus far, no selective MuSK inhibitors have been described.

Further reading on Type IX RTKs: MuSKHerbstR (2020) MuSk function during health and disease. Neurosci Lett
716: 13467631811897
10.1016/j.neulet.2019.134676KeritamO
 (2024) A clinical perspective on muscle specific kinase antibody positive myasthenia gravis. Front Immunol
15: 150248039703505
10.3389/fimmu.2024.1502480PMC11655327

## Type X RTKs: HGF (hepatocyte growth factor) receptor family

Catalytic receptors → Receptor kinases → TK: Tyrosine kinase → Receptor tyrosine kinases (RTKs) → Type X RTKs: HGF (hepatocyte growth factor) receptor family

### Overview:

The hepatocyte growth factor (HGF) receptor family - MET and Ron - regulate maturation of the liver in the embryo, as well as having roles in the adult, for example, in the innate immune system. HGF is synthesized as a single gene product, which is post-translationally processed to yield a heterodimer linked by a disulphide bridge. The maturation of HGF is enhanced by a serine protease, HGF activating complex, and inhibited by HGF-inhibitor 1 (*SPINT1*, O43278), a serine protease inhibitor. MST1, the ligand of Ron, is two disulphide-linked peptide chains generated by proteolysis of a single gene product.

**Table:** 

Nomenclature	MET proto-oncogene, receptor tyrosine kinase	macrophage stimulating 1 receptor
Common abbreviation	MET	Ron
HGNC, UniProt	*MET*, P08581	*MST1R*, Q04912
EC number	2.7.10.1	2.7.10.1
Endogenous ligands	hepatocyte growth factor (*HGF*, P14210)	macrophage stimulating protein 1 (*MST1*, P09603)
Inhibitors	capmatinib (pIC_50_ 9.9) [169], SGX-523 (p*K*_d_ 9.7) [51], cabozantinib (pIC_50_ 8.9) [293]	BMS-777607 (pIC_50_ 8.7) [237]
Selective inhibitors	SGX-523 (pIC_50_ 8.4) [29]	–

### Comments:

PF04217903 is a selective Met tyrosine kinase inhibitor [48]. SU11274 is an inhibitor of the HGF receptor [233], with the possibility of further targets [8].

## Type XI RTKs: TAM (TYRO3- , AXL- and MER-TK) receptor family

Catalytic receptors → Receptor kinases → TK: Tyrosine kinase → Receptor tyrosine kinases (RTKs) → Type XI RTKs: TAM (TYRO3-, AXL- and MER-TK) receptor family

### Overview:

The TAM receptor family, named from the first letter of each of its constituents, respond to growth arrest specific protein 6 (*GAS6*, Q14393) and protein S (*PROS1*, P07225). These ligands are secreted plasma proteins which undergo vitamin K-dependent post-translational modifications generating carboxyglutamate-rich domains which are able to bind to negatively-charged surfaces of apoptotic cells. Members of this RTK family represented a novel structural motif, when originally sequenced.

**Table:** 

Nomenclature	AXL receptor tyrosine kinase	TYRO3 protein tyrosine kinase	MER proto-oncogene, tyrosine kinase
Common abbreviation	Axl	Tyro3	Mer
HGNC, UniProt	*AXL*, P30530	*TYRO3*, Q06418	*MERTK*, Q12866
EC number	2.7.10.1	2.7.10.1	2.7.10.1
Endogenous ligands	growth arrest specific protein 6 (*GAS6*, Q14393) [200], protein S (*PROS1*, P07225) [255]	growth arrest specific protein 6 (*GAS6*, Q14393) [200], protein S (*PROS1*, P07225) [255]	growth arrest specific protein 6 (*GAS6*, Q14393) [200]

### Comments:

AXL tyrosine kinase inhibitors have been described [193].

Further reading on Type XI RTKs: TAM (TYRO3-, AXL- and MER-TK) receptor familyLemkeG (2013) Biology of the TAM receptors. Cold Spring Harb Perspect Biol
5: a00907624186067
10.1101/cshperspect.a009076PMC3809585

## Type XII RTKs: TIE family of angiopoietin receptors

Catalytic receptors → Receptor kinases → TK: Tyrosine kinase → Receptor tyrosine kinases (RTKs) → Type XII RTKs: TIE family of angiopoietin receptors

### Overview:

The TIE family were initially associated with formation of blood vessels (angiogenesis). Endogenous ligands are angiopoietin-1 (*ANGPT1*, Q15389), angiopoietin-2 (*ANGPT2*, O15123), and angiopoietin-4 (*ANGPT4*, Q9Y264). Angiopoietin-2 (*ANGPT2*, O15123) appears to act as an endogenous antagonist of angiopoietin-1 function, thus blocking TIE2-mediated signalling. Due to roles in blood vessel formation, monoclonal antibodies are being developed against the ligand Ang2 (cyamemazine) or a bispecific antibody against VEGF and Ang2 (faricimab) for the treatment of ocular diseases.

**Table:** 

Nomenclature	tyrosine kinase with immunoglobulin like and EGF like domains 1	TEK receptor tyrosine kinase
Common abbreviation	TIE1	TIE2
HGNC, UniProt	*TIE1*, P35590	*TEK*, Q02763
EC number	2.7.10.1	2.7.10.1
Endogenous ligands	–	angiopoietin-1 (*ANGPT1*, Q15389), angiopoietin-4 (*ANGPT4*, Q9Y264)

Further reading on Type XII RTKs: TIE family of angiopoietin receptorsAkwiiRG
 (2021) Targeting the Angiopoietin/Tie Pathway: Prospects for Treatment of Retinal and Respiratory Disorders: Drugs. 81: 1731–174934586603
10.1007/s40265-021-01605-yPMC8479497KhananiAM
 (2021) Angiopoietins as Potential Targets in Management of Retinal Disease. Clin Ophthalmol
15: 3747–375534511878
10.2147/OPTH.S231801PMC8427682SaharinenP
 (2017) Therapeutic targeting of the angiopoietin-TIE pathway. Nat Rev Drug Discov
16: 635–66128529319
10.1038/nrd.2016.278

## Type XIII RTKs: Ephrin receptor family

Catalytic receptors → Receptor kinases → TK: Tyrosine kinase → Receptor tyrosine kinases (RTKs) → Type XIII RTKs: Ephrin receptor family

### Overview:

Ephrin receptors are a family of 15 RTKs - the largest family of RTKs - with two identified subfamilies (EphA and EphB), which have a role in the regulation of neuronal development, cell migration, patterning and angiogenesis. Their ligands are membrane-associated proteins, thought to be glycosylphosphatidylinositol-linked for EphA (ephrin-A1 (*EFNA1*, P20827), ephrin-A2 (*EFNA2*, O43921), ephrin-A3 (*EFNA3*, P52797), ephrin-A4 (*EFNA4*, P52798) and ephrin-A5 (*EFNA5*, P52803)) and transmembrane proteins for Ephrin B (ENSFM00250000002014: ephrin-B1 (*EFNB1*, P98172), ephrin-B2 (*EFNB2*, P52799) and ephrin-B3 (*EFNB3*, Q15768)). Ephrin-A3 and ephrin-B3 have also been shown to interact with heparan sulphate proteoglycans [164].

**Table:** 

Nomenclature	EPH receptor A1	EPH receptor A2	EPH receptor A3	EPH receptor A4	EPH receptor A5	EPH receptor A6	EPH receptor A7
Common abbreviation	EphA1	EphA2	EphA3	EphA4	EphA5	EphA6	EphA7
HGNC, UniProt	*EPHA1*, P21709	*EPHA2*, P29317	*EPHA3*, P29320	*EPHA4*, P54764	*EPHA5*, P54756	*EPHA6*, Q9UF33	*EPHA7*, Q15375
EC number	2.7.10.1	2.7.10.1	2.7.10.1	2.7.10.1	2.7.10.1	2.7.10.1	2.7.10.1

**Table:** 

Nomenclature	EPH receptor A8	EPH receptor A10	EPH receptor B1	EPH receptor B2	EPH receptor B3	EPH receptor B4	EPH receptor B6
Common abbreviation	EphA8	EphA10	EphB1	EphB2	EphB3	EphB4	EphB6
HGNC, UniProt	*EPHA8*, P29322	*EPHA10*, Q5JZY3	*EPHB1*, P54762	*EPHB2*, P29323	*EPHB3*, P54753	*EPHB4*, P54760	*EPHB6*, O15197
EC number	2.7.10.1	2.7.10.1	2.7.10.1	2.7.10.1	2.7.10.1	2.7.10.1	2.7.10.1
Inhibitors	–	–	compound 66 (pIC_50_ 9) [149]	–	–	tesevatinib (pIC_50_ 8.9) [86]	–

Further reading on Type XIII RTKs: Ephrin receptor familyLisabethEM
 (2013) Eph receptor signaling and ephrins. Cold Spring Harb Perspect Biol
510.1101/cshperspect.a009159PMC375371424003208RasoolD
 (2024) Master regulators of neurogenesis: the dynamic roles of Ephrin receptors across diverse cellular niches. Transl Psychiatry
14: 46239505843
10.1038/s41398-024-03168-4PMC11541728

## Type XIV RTKs: RET

Catalytic receptors → Receptor kinases → TK: Tyrosine kinase → Receptor tyrosine kinases (RTKs) → Type XIV RTKs: RET

### Overview:

The RET (REarranged during Transfection) receptor is a transmembrane tyrosine kinase enzyme enzyme. RET forms a complex with members of the GPI-linked GDNF family receptors (GFR) to respond to GDNF family ligands, including glial cell-derived neurotrophic factors including glial cell-derived neurotrophic factor GDNF (*GDNF*, P39905) (211 aa); neurturin (*NRTN*, Q99748) (197 aa); artemin (*ARTN*, Q5T4W7) (237 aa) and persephin (*PSPN*, O60542) (156 aa). RET also forms a complex with GFRAL, which is activated by growth differentiation factor 15 (*GDF15*, Q99988) (GDF15) [197, 295]. RET is involved in neural crest development. Loss of function mutations lead to Hirschprung’s disease, while gain of function mutations lead to multiple endocrine neoplasias type 2A and 2B. There are isoforms - RET51 and RET9 - with distinct signalling properties.

**Table:** 

Nomenclature	ret proto-oncogene
Common abbreviation	Ret
HGNC, UniProt	*RET*, P07949
EC number	2.7.10.1
Inhibitors	tamatinib (pIC_50_ 8.3) [40], selpercatinib (pIC_50_ 7.8) [6]

### Comments:

A number of tyrosine kinase inhibitors targeting RET have been described [66], including selpercatinib, a RET inhibitor FDA-approved for non-small-cell lung cancer [59].

Further reading on Type XIV RTKs: RETIbáñezCF
 (2020) RET-independent signaling by GDNF ligands and GFRα receptors. Cell Tissue Res
382: 71–8232737575
10.1007/s00441-020-03261-2PMC7529620IbáñezCF
 (2017) Biology of GDNF and its receptors - Relevance for disorders of the central nervous system. Neurobiol Dis
97: 80–8926829643
10.1016/j.nbd.2016.01.021SidorovaYA
 (2020) Small Molecules and Peptides Targeting Glial Cell Line-Derived Neurotrophic Factor Receptors for the Treatment of Neurodegeneration. Int J Mol Sci
2110.3390/ijms21186575PMC755478132911810WangD
 (2021) GDF15: emerging biology and therapeutic applications for obesity and cardiometabolic disease. Nat Rev Endocrinol
17: 592–60734381196
10.1038/s41574-021-00529-7

## Type XV RTKs: RYK

Catalytic receptors → Receptor kinases → TK: Tyrosine kinase → Receptor tyrosine kinases (RTKs) → Type XV RTKs: RYK

### Overview:

The ’related to tyrosine kinase receptor’ (Ryk) is structurally atypical of the family of RTKs, particularly in the activation and ATP-binding domains, lacking kinase activity akin to ROR1/2.

Similarly, however, there is evidence that RTK is involved in Wnt signalling [222].

**Table:** 

Nomenclature	receptor like tyrosine kinase
Common abbreviation	RYK
HGNC, UniProt	*RYK*, P34925
EC number	2.7.10.1

### Comments:

Thus far, no selective RYK inhibitors have been described.

Further reading on Type XV RTKs: RYKGreenJ
 (2014) The role of Ryk and Ror receptor tyrosine kinases in Wnt signal transduction. Cold Spring Harb Perspect Biol
610.1101/cshperspect.a009175PMC394123624370848RoyJP
 (2018) The biochemistry, signalling and disease relevance of RYK and other WNT-binding receptor tyrosine kinases. Growth Factors
36: 15–4029806777
10.1080/08977194.2018.1472089

## Type XVI RTKs: DDR (collagen receptor) family

Catalytic receptors → Receptor kinases → TK: Tyrosine kinase → Receptor tyrosine kinases (RTKs) → Type XVI RTKs: DDR (collagen receptor) family

### Overview:

The discoidin domain receptors DDR1 and DDR2 are structurally related receptor tyrosine kinases that function as collagen receptors. The 28 different collagens form the most abundant protein family in man. Collagens are found in the extracellular matrix and are generally deposited there in the form of supramolecular assemblies arranged from triple-helical rope-like structural units. In man, the main collagens include COL1A1 (*COL1A1*, P02452), COL2A1 (*COL2A1*, P02458), COL3A1 (*COL3A1*, P02461) and COL4A1 (*COL4A1*, P02462).

**Table:** 

Nomenclature	discoidin domain receptor tyrosine kinase 1	discoidin domain receptor tyrosine kinase 2
Common abbreviation	DDR1	DDR2
HGNC, UniProt	*DDR1*, Q08345	*DDR2*, Q16832
EC number	2.7.10.1	2.7.10.1
Selective inhibitors	DDR1 inhibitor 7rh (pIC_50_ 8.2) [81], DDR-IN-1 (pIC_50_ 7) [138]	–
Antibodies	mAb PRTH-101 (Inhibition) (p*K*_d_ 9.4) [167], mAb 3E3 (Inhibition) [32]	–

### Comments:

Other collagen receptors include four collagen-binding integrins of the β1 integrin family (ITGB1, P05556), with the following α subunits: α1 (ITGA1, P56199), α2 (ITGA2, P17301), α10 (ITGA10, O75578) or α11 (ITGA11, Q9UKX5). Additional collagen receptors include, glycoprotein VI (GP6, Q9HCN6), leukocyte-associated immunoglobulin-like receptor 1 (LAIR1, Q6GTX8), leukocyte-associated immunoglobulin-like receptor 2 (LAIR2, Q6ISS4) and osteoclast-associated immunoglobulin-like receptor (OSCAR, Q8IYS5).

The tyrosine kinase inhibitors of DDR, imatinib and nilotinib, were identified from proteomic analysis [52].

## Type XVII RTKs: ROS receptors

Catalytic receptors → Receptor kinases → TK: Tyrosine kinase → Receptor tyrosine kinases (RTKs) → Type XVII RTKs: ROS receptors

### Overview:

The *ROS1* gene encodes a receptor tyrosine kinase, whose endogenous substrate and normal physiological functions are not fully determined, however there is evidence that neural epidermal growth factor-like like 2 (*NELL2*; Q99435) is an agonist for ROS1. Aberrant expression and mutated forms of ROS1 are drivers of malignant transformation in number of tumour types.

**Table:** 

Nomenclature	c-ros oncogene 1, receptor tyrosine kinase
Common abbreviation	ROS
HGNC, UniProt	*ROS1*, P08922
EC number	2.7.10.1

### Comments:

Crizotinib and cabozantinib are Type I and Type II tyrosine kinase inhibitors, respectively, targeting ROS1 for the treatment of cancer.

Further reading on Type XVII RTKs: ROS receptorsDrilonA
 (2021) ROS1-dependent cancers - biology, diagnostics and therapeutics. Nat Rev Clin Oncol
18: 35–5532760015
10.1038/s41571-020-0408-9PMC8830365

## Type XVIII RTKs: LMR family

Catalytic receptors → Receptor kinases → TK: Tyrosine kinase → Receptor tyrosine kinases (RTKs) → Type XVIII RTKs: LMR family

### Overview:

The lemur tail kinase (LMR) family are are unusual amongst the RTKs in possessing a short extracellular domain and extended intracellular domain (hence the ’Lemur’ name reflecting the long tail). LMR1 was identified as a potential marker of apoptosis [82], giving rise to the name AATYK (Apoptosis-Associated Tyrosine Kinase); while over-expression induces differentiation in neuroblastoma cells [224]. The LMTK/LMR family have since been identified to have serine/threonine kinase activity, as opposed to tyrosine kinase [281].

**Table:** 

Nomenclature	apoptosis associated tyrosine kinase	lemur tyrosine kinase 2	lemur tyrosine kinase 3
Common abbreviation	Lmr1	Lmr2	Lmr3
HGNC, UniProt	*AATK*, Q6ZMQ8	*LMTK2*, Q8IWU2	*LMTK3*, Q96Q04
EC number	2.7.11.1	2.7.11.1	2.7.11.1

### Comments:

As yet no selective inhibitors of the LMR family have been described.

Further reading on Type XVIII RTKs: LMR familyMórotzGM
 (2024) A revised nomenclature for the lemur family of protein kinases. Commun Biol
7: 5738191649
10.1038/s42003-023-05671-8PMC10774328WendlerF
 (2021) The LMTK-family of kinases: Emerging important players in cell physiology and pathogenesis. Biochim Biophys Acta Mol Basis Dis
1867: 16537230597196
10.1016/j.bbadis.2018.12.023

## Type XIX RTKs: Leukocyte tyrosine kinase (LTK) receptor family

Catalytic receptors → Receptor kinases → TK: Tyrosine kinase → Receptor tyrosine kinases (RTKs) → Type XIX RTKs: Leukocyte tyrosine kinase (LTK) receptor family

### Overview:

The LTK family are receptor tyrosine kinases activated by FAM150A/B ligands [97, 225], also known as AUG-β and AUG-α, respectively. LTK is subject to tissue-specific splice variation, which appears to generate products in distinct subcellular locations. ALK fusions resulting from gene translocations and rearrangements are associated with many types of cancer, including large cell lymphomas, inflammatory myofibrilastic tumours and non-small cell lung cancer [180].

**Table:** 

Nomenclature	leukocyte receptor tyrosine kinase	ALK receptor tyrosine kinase
Common abbreviation	LTK	ALK
HGNC, UniProt	*LTK*, P29376	*ALK*, Q9UM73
EC number	2.7.10.1	2.7.10.1
Inhibitors	–	GSK-1838705A (pIC_50_ 9.3) [231], compound 8e (pIC_50_ 9.1) [118], NVP-TAE684 (pK_d_ 9) [51], compound 25b (pIC_50_ 8.7) [88]
Selective inhibitors	–	ceritinib (pIC_50_ 9.7) [180]
Endogenous agonists	FAM150A (ALKAL1) (Activation) [97], FAM150B (ALKAL2) (Activation) [97]	FAM150B (ALKAL2) (Activation) [97]

## Type XX RTKs: STYK1

Catalytic receptors → Receptor kinases → TK: Tyrosine kinase → Receptor tyrosine kinases (RTKs) → Type XX RTKs: STYK1

### Overview:

Similar to the LMR RTK family, STYK1 has a truncated extracellular domain, but also displays a relatively short intracellular tail beyond the split kinase domain. Also known as NOK, STYK1 has been linked to EGFR signalling [58, 62].

**Table:** 

Nomenclature	serine/threonine/tyrosine kinase 1
Common abbreviation	STYK1
HGNC, UniProt	*STYK1*, Q6J9G0
EC number	2.7.10.2

### Comments:

As yet, no selective inhibitors of STYK1 have been described.

Further reading on Receptor tyrosine kinases (RTKs)Álvarez-AznarA
 (2017) VEGF Receptor Tyrosine Kinases: Key Regulators of Vascular Function. Curr Top Dev Biol
123: 433–48228236974
10.1016/bs.ctdb.2016.10.001BergeronJJ
 (2016) Spatial and Temporal Regulation of Receptor Tyrosine Kinase Activation and Intracellular Signal Transduction. Annu Rev Biochem
85: 573–9727023845
10.1146/annurev-biochem-060815-014659CarvalhoS
 (2016) Immunotherapy of cancer: from monoclonal to oligoclonal cocktails of anti-cancer antibodies: IUPHAR Review 18. Br J Pharmacol
173: 1407–2426833433
10.1111/bph.13450PMC4831314De SilvaDM
 (2017) Targeting the hepatocyte growth factor/Met pathway in cancer. Biochem Soc Trans
45: 855–87028673936
10.1042/BST20160132EklundL
 (2017) Angiopoietin-Tie signalling in the cardiovascular and lymphatic systems. Clin Sci
131: 87–10310.1042/CS20160129PMC514695627941161KatayamaR (2017) Therapeutic strategies and mechanisms of drug resistance in anaplastic lymphoma kinase (ALK)-rearranged lung cancer. Pharmacol Ther
177: 1–828185914
10.1016/j.pharmthera.2017.02.015KazlauskasA (2017) PDGFs and their receptors. Gene
614: 1–728267575
10.1016/j.gene.2017.03.003PMC6728141KeEE
 (2016) EGFR as a Pharmacological Target in EGFR-Mutant Non-Small-Cell Lung Cancer: Where Do We Stand Now?
Trends Pharmacol Sci
37: 887–90327717507
10.1016/j.tips.2016.09.003KuwanoM
 (2016) Overcoming drug resistance to receptor tyrosine kinase inhibitors: Learning from lung cancer. Pharmacol Ther
161: 97–11027000770
10.1016/j.pharmthera.2016.03.002LeeDH. (2017) Treatments for EGFR-mutant non-small cell lung cancer (NSCLC): The road to a success, paved with failures. Pharmacol Ther
174: 1–2128167215
10.1016/j.pharmthera.2017.02.001NelsonKN
 (2017) Receptor Tyrosine Kinases: Translocation Partners in Hematopoietic Disorders. Trends Mol Med
23: 59–7927988109
10.1016/j.molmed.2016.11.002SimonsM
 (2016) Mechanisms and regulation of endothelial VEGF receptor signalling. Nat Rev Mol Cell Biol
17: 611–2527461391
10.1038/nrm.2016.87StrickerS
 (2017) ROR-Family Receptor Tyrosine Kinases. Curr Top Dev Biol
123: 105–14228236965
10.1016/bs.ctdb.2016.09.003TanAC
 (2017) Exploiting receptor tyrosine kinase co-activation for cancer therapy. Drug Discov Today
22: 72–8427452454
10.1016/j.drudis.2016.07.010PMC5346155

## Receptor serine/threonine kinase (RSTK) family

Catalytic receptors → Receptor kinases → TKL: Tyrosine kinase-like → Receptor serine/threonine kinase (RSTK) family

### Overview:

Receptor serine/threonine kinases (RSTK), EC 2.7.11.30, respond to particular cytokines, the transforming growth factor β (TGFβ) and bone morphogenetic protein (BMP) families, and may be divided into two subfamilies on the basis of structural similarities. Agonist binding initiates formation of a cell-surface complex of type I and type II RSTK, possibly heterotetrameric, where both subunits express serine/threonine kinase activity. The type I receptor serine/threonine kinases are also known as activin receptors or activin receptor-like kinases, ALKs, for which a systematic nomenclature has been proposed (ALK1-7). The type II protein phosphorylates the kinase domain of the type I partner (sometimes referred to as the signal propagating subunit), causing displacement of the protein partners, such as the FKBP12 FK506-binding protein FKBP1A (P62942) and allowing the binding and phosphorylation of particular members of the Smad family. These migrate to the nucleus and act as complexes to regulate gene transcription. Type III receptors, sometimes called co-receptors or accessory proteins, regulate the signalling of the receptor complex, in either enhancing (for example, presenting the ligand to the receptor) or inhibitory manners. TGFβ family ligand signalling may be inhibited by endogenous proteins, such as follistatin (FST, P19883), which binds and neutralizes activins to prevent activation of the target receptors. Endogenous agonists, approximately 30 in man, are often described as paracrine messengers acting close to the source of production. They are characterized by six conserved cysteine residues and are divided into two subfamilies on the basis of sequence comparison and signalling pathways activated, the TGFβ/activin/nodal subfamily and the BMP/GDF (growth/differentiation factor)/MIS (Müllerian inhibiting substance) subfamily. Ligands active at RSTKs appear to be generated as large precursors which undergo complex maturation processes [159]. Some are known to form disulphide-linked homo- and/or heterodimeric complexes. Thus, inhibins are α subunits linked to a variety of β chains, while activins are combinations of β subunits.

## Type I receptor serine/threonine kinases

Catalytic receptors → Receptor kinases → TKL: Tyrosine kinase-like → Receptor serine/threonine kinase (RSTK) family → Type I receptor serine/threonine kinases

### Overview:

The type I receptor serine/threonine kinases are also known as activin receptors or activin receptor-like kinases, ALKs, for which a systematic nomenclature has been proposed (ALK1-7). These receptors are responsible for signalling in response to transforming growth factor beta (TGFβ) [170]. These act as a heterodimer, where Type I RSTKs are phosphorylated by Type II RSTKs.

**Table:** 

Nomenclature	activin A receptor type IL	activin A receptor type 1	bone morphogenetic protein receptor type IA	activin A receptor type 1B	transforming growth factor beta receptor 1	bone morphogenetic protein receptor type IB	activin A receptor type 1C
Common abbreviation	ALK1	ALK2	BMPR1A	ALK4	TGFBR1	BMPR1B	ALK7
HGNC, UniProt	*ACVRL1*, P37023	*ACVR1*, Q04771	*BMPR1A*, P36894	*ACVR1B*, P36896	*TGFBR1*, P36897	*BMPR1B*, O00238	*ACVR1C*, Q8NER5
EC number	2.7.11.30	2.7.11.30	2.7.11.30	2.7.11.30	2.7.11.30	2.7.11.30	2.7.11.30
Inhibitors	–	ML347 (pIC_50_ 7.5) [_64_]	–	–	LY2109761 (p*K*_i_ 7.4) [188], compound 15b (pIC_50_ 7.1) [158]	–	–
Selective inhibitors	–	–	–	vactosertib (pIC_50_ 7.9) [130]	vactosertib (pIC_50_ 8) [130]	–	–

Further reading on Type I receptor serine/threonine kinasesBatlleE
 (2019) Transforming Growth Factor-β Signaling in Immunity and Cancer. Immunity
50: 924–94030995507
10.1016/j.immuni.2019.03.024PMC7507121

## Type II receptor serine/threonine kinases

Catalytic receptors → Receptor kinases → TKL: Tyrosine kinase-like → Receptor serine/threonine kinase (RSTK) family → Type II receptor serine/threonine kinases

### Overview:

Type II protein receptor serine/threonine kinases interact with transforming growth factor beta (TGFβ), bone morphogenic protein (BMP) or MÀllerian inhibiting substrate (MIS). Type II RSTKs then phosphorylate the kinase domain of their Type I RSTK partner - sometimes referred to as the signal propagating unit. This causes displacement of protein partners, such as the FKBP12 FK506-binding protein *FKBP1A* (P62942) and allowing the binding and phosphorylation of particular members of the Smad family.

**Table:** 

Nomenclature	activin A receptor type 2A	activin A receptor type 2B	anti-Mullerian hormone receptor type 2	bone morphogenetic protein receptor type 2	transforming growth factor beta receptor 2
Common abbreviation	ActR2	ActR2B	MISR2	BMPR2	TGFBR2
HGNC, UniProt	*ACVR2A*, P27037	*ACVR2B*, Q13705	*AMHR2*, Q16671	*BMPR2*, Q13873	*TGFBR2* P37173
EC number	2.7.11.30	2.7.11.30	2.7.11.30	2.7.11.30	2.7.11.30
Antibodies	–	bimagrumab (Binding) (p*K_d_* 11.8) [14]	–	–	–

Further reading on Type II receptor serine/threonine kinasesBatlleE
 (2019) Transforming Growth Factor-β Signaling in Immunity and Cancer. Immunity
50: 924–94030995507
10.1016/j.immuni.2019.03.024PMC7507121

## Type III receptor serine/threonine kinases

Catalytic receptors → Receptor kinases → TKL: Tyrosine kinase-like → Receptor serine/threonine kinase (RSTK) family → Type III receptor serine/threonine kinases

### Overview:

Type III serine/threonine kinase is often referred to as an accessory protein. While it has no known enzymatic activity, it can regulate the signalling of RSTKs [115, 148].

**Table:** 

Nomenclature	transforming growth factor beta receptor 3
Common abbreviation	TGFBR3
HGNC, UniProt	*TGFBR3*, Q03167
Comments	Also known as betaglycan.

## RSTK functional heteromers

Catalytic receptors → Receptor kinases → TKL: Tyrosine kinase-like → Receptor serine/threonine kinase (RSTK) family → RSTK functional heteromers

### Overview:

For the receptors listed on this page, the exact combination of subunits forming the functional heteromeric receptors is unknown.

**Table:** 

Nomenclature	Transforming growth factor β receptor	Bone morphogenetic protein receptors
Subunits	transforming growth factor beta receptor 1 (Type I), transforming growth factor beta receptor 2 (Type II), transforming growth factor beta receptor 3 (Type III)	bone morphogenetic protein receptor type IA (Type I), bone morphogenetic protein receptor type IB (Type I), activin A receptor type 1 (Type I), activin A receptor type IL (Type I), bone morphogenetic protein receptor type 2 (Type II), activin A receptor type 2A (Type II), activin A receptor type 2B (Type II)
Coupling	Smad2, Smad3 [196, 245]	Smad1, Smad5, Smad8 [196, 245]
Endogenous agonists	TGFβ1 (*TGFB1*, P01137), TGFβ2 (*TGFB2*, P61812), TGFβ3 (*TGFB3*, P10600)	BMP-10 (*BMP10*, O95393), BMP-2 (*BMP2*, P12643), BMP-4 (*BMP4*, P12644), BMP-5 (*BMP5*, P22003), BMP-6 (*BMP6*, P22004), BMP-7 (*BMP7*, P18075), BMP-8A (*BMP8A*, Q7Z5Y6), BMP-8B (*BMP8B*, P34820), BMP-9 (*GDF2*, Q9UK05)

**Table:** 

Nomenclature	Growth/differentiation factor receptors	Activin receptors	Anti-Müllerian hormone receptors
Subunits	bone morphogenetic protein receptor type IA (Type I), bone morphogenetic protein receptor type IB (Type I), activin A receptor type 1B (Type I), activin A receptor type 1C (Type I), transforming growth factor beta receptor 1 (Type I), bone morphogenetic protein receptor type 2 (Type II), activin A receptor type 2A (Type II), activin A receptor type 2B (Type II)	activin A receptor type 1B (Type I), activin A receptor type 1C (Type I), activin A receptor type 2A (Type II), activin A receptor type 2B (Type II)	bone morphogenetic protein receptor type IA (Type I), bone morphogenetic protein receptor type IB (Type I), activin A receptor type 1 (Type I), anti-Mullerian hormone receptor type 2 (Type II)
Coupling	Smad1, Smad5, Smad8 [196, 245]	Smad2, Smad3 [245]	Smad1, Smad5, Smad8 [196, 245]
Endogenous agonists	growth/differentiation factor-1 (*GDF1*, P27539), growth/differentiation factor-10 (*GDF10*, P55107), growth/diffferentiation factor-3 (*GDF3*, Q9NR23), growth/differentiation factor-7 (*GDF7*, Q7Z4P5), growth/differentiation factor-9 (*GDF9*, O60383)	activin A (*INHBA*, P08476), activin AB (*INHBA*INHBB, P08476P09529), activin B (*INHBB*, P09529), inhibin A (*INHA*INHBA, P05111P08476)	Müllerian inhibiting substance (*AMH*, P03971)
Comments	–	Activin receptors are heteromeric complexes comprising activin receptor type I and type II subunits.	–

Further reading on RSTK functional heteromersBatlleE
 (2019) Transforming Growth Factor-β Signaling in Immunity and Cancer. Immunity
50: 924–94030995507
10.1016/j.immuni.2019.03.024PMC7507121

### Comments on Receptor serine/threonine kinase (RSTK) family:

A number of endogenous inhibitory ligands have been identified for RSTKs, including BMP-3 (*BMP3*, P12645), inhibin α (*INHA*, P05111), inhibin βC (*INHBC*, P55103) and inhibin βE (*INHBE*, P58166).

An appraisal of small molecule inhibitors of TGFβ and BMP signalling concluded that TGFβ pathway inhibitors were more selective than BMP signalling inhibitors [274]. The authors confirmed the selectivity of TGF-beta RI inhibitor III to inhibit TGFβ signalling through ALK4, ALK5, ALK7 [49]. Dorsomorphin inhibits BMP signalling through ALK2 and ALK3, it also inhibits AMP kinase [305].

**Smads** were identified as mammalian orthologues of Drosophila genes termed “mothers against decapentaplegic” and may be divided into Receptor-regulated Smads (R-Smads, including Smad1, Smad2, Smad3, Smad5 and Smad8), Co-mediated Smad (Co-Smad, Smad4) and Inhibitory Smads (I-Smad, Smad6 and Smad7). R-Smads form heteromeric complexes with Co-Smad. I-Smads compete for binding of R-Smad with both receptors and Co-Smad.

**Table:** 

Nomenclature	HGNC gene symbol	Uniprot ID	Other names
Smad1	*SMAD1*	Q15797	JV4-1, MADH1, MADR1
Smad2	*SMAD2*	Q15796	JV18-1, MADH2, MADR2
Smad3	*SMAD3*	P84022	HsT17436, JV15-2, MADH3
Smad4	*SMAD4*	Q13485	DPC4, MADH4
Smad5	*SMAD5*	Q99717	Dwfc, JV5-1, MADH5
Smad6	*SMAD6*	O43541	HsT17432, MADH6, MADH7
Smad7	*SMAD7*	O15105	MADH7, MADH8
Smad8	*SMAD9*	O15198	MADH6, MADH9

Further reading on Receptor serine/threonine kinase (RSTK) familyBudiEH
 (2017) Transforming Growth Factor-β Receptors and Smads: Regulatory Complexity and Functional Versatility. Trends Cell Biol
27: 658–67228552280
10.1016/j.tcb.2017.04.005ChenW
 (2016) Immunoregulation by members of the TGFβ superfamily. Nat Rev Immunol
16: 723–74027885276
10.1038/nri.2016.112HegerJ
 (2016) Molecular switches under TGFβ signalling during progression from cardiac hypertrophy to heart failure. Br J Pharmacol
173: 3–1426431212
10.1111/bph.13344PMC4813390LuoJY
 (2015) Regulators and effectors of bone morphogenetic protein signalling in the cardiovascular system. J Physiol (Lond.)
593: 2995–301125952563
10.1113/JP270207PMC4532521MaciasMJ
 (2015) Structural determinants of Smad function in TGF-β signaling. Trends Biochem Sci
40: 296–30825935112
10.1016/j.tibs.2015.03.012PMC4485443MorrellNW
 (2016) Targeting BMP signalling in cardiovascular disease and anaemia. Nat Rev Cardiol
13: 106–2026461965
10.1038/nrcardio.2015.156PMC4886232NeuzilletC
 (2015) Targeting the TGFβ pathway for cancer therapy. Pharmacol Ther
147: 22–3125444759
10.1016/j.pharmthera.2014.11.001van der KraanPM. (2017) The changing role of TGFβ in healthy, ageing and osteoarthritic joints. Nat Rev Rheumatol
13: 155–16328148919
10.1038/nrrheum.2016.219

## Receptor tyrosine phosphatase (RTP) family

Catalytic receptors → Receptor tyrosine phosphatase (RTP) family

### Overview:

Receptor tyrosine phosphatases (RTP)- also referred to as receptor-type tyrosine-protein phosphatases (PTPR) - are cell-surface proteins with a single TM region and intracellular phosphatase activity at phosphorylated tyrosine residues. There are 20 family members of classic RPTPs. Many family members exhibit constitutive activity upon heterologous expression, dephosphorylating intracellular targets such as Src tyrosine kinase (SRC) to activate signalling cascades. Family members bind components of the extracellular matrix or cell-surface proteins indicating a role in intercellular communication.

**Table:** 

Nomenclature	RTP Type A	RTP Type B	RTP Type C	RTP Type D	RTP Type E	RTP Type F	RTP Type G
HGNC, UniProt	*PTPRA*, P18433	*PTPRB*, P23467	*PTPRC*, P08575	*PTPRD*, P23468	*PTPRE*, P23469	*PTPRF* P10586	*PTPRG*, P23470
Putative endogenous ligands	–	–	galectin-1 (*LGALS1*, P09382) [275]	netrin-G3 ligand (*LR-RC4B*, Q9NT99) [147]	–	netrin-G3 ligand (*LR-RC4B*, Q9NT99) [147]	contactin-3 (*CNTN3*, Q9P232), contactin-4 (*CNTN4*, Q8IWV2), contactin-5 (*CNTN5*, O94779), contactin-6 (CNTN6, Q9UQ52) [22]
Inhibitors	–	–	–	–	–	illudalic acid (pIC_50_ 5.9) [163]	compound 1 (p*K*_i_ 5.6) [244]
Comments	–	–	–	PTPRD is also known as PTPS	–	PTPRF is also known as LAR	–

**Table:** 

Nomenclature	RTP Type H	RTP Type J	RTP Type K	RTP Type M	RTP Type N	RTP Type N2	RTP Type O
HGNC, UniProt	*PTPRH* Q9HD43	*PTPRJ* Q12913	*PTPRK*, Q15262	*PTPRM* P28827	*PTPRN*, Q16849	*PTPRN2*, Q92932	*PTPRO*, Q16827
Putative endogenous ligands	–	–	galectin-3 (*LGALS3*, P17931), galectin-3 binding protein (*LGALS3BP*, Q08380) [140]	–	–	–	–
Activators	–	QM107 (Binding)	–	–	–	–	–
Inhibitors	–	–	–	compound 8a (pIC_50_ 5.2) [106]	–	–	–
Comments	–	PTPRJ (CD148) is a therapeutic target for the treament of human diseases [157].	–	–	–	–	–

**Table:** 

Nomenclature	RTP Type Q	RTP Type R	RTP Type S	RTP Type T	RTP Type U	RTP Type Z1
HGNC, UniProt	*PTPRQ*, Q9UMZ3	*PTPRR*, Q15256	*PTPRS*, Q13332	*PTPRT* O14522	*PTPRU* Q92729	*PTPRZ1*, P23471
Putative endogenous ligands	–	–	chondroitin sulphate proteoglycan 3 (*NCAN*, O14594), netrin-G3 ligand (*LRRC4B*, Q9NT99) [147, 243]	–	–	contactin-1 (*CNTN1*, Q12860), pleiotrophin (*PTN*, C9JR52) (acts as a negative regulator) [22, 190]
Inhibitors	–	–	compound 7b (pIC_50_ 5.4) [98], 7-BIA (pIC_50_ 4.4) [270]	–	–	–

Further reading on Receptor tyrosine phosphatase (RTP) familyPapadimitriouE
 (2016) Pleiotrophin and its receptor protein tyrosine phosphatase beta/zeta as regulators of angiogenesis and cancer. Biochim Biophys Acta
1866: 252–26527693125
10.1016/j.bbcan.2016.09.007StanfordSM
 (2017) Targeting Tyrosine Phosphatases: Time to End the Stigma. Trends Pharmacol Sci
38: 524–54028412041
10.1016/j.tips.2017.03.004PMC5494996

## Tumour necrosis factor (TNF) receptor family

Catalytic receptors → Tumour necrosis factor (TNF) receptor family

### Overview:

The TNF receptor family has at least 29 genes, with diverse roles in cell death, inflammation and development. Dysregulated TNFR signalling is associated with many inflammatory disorders, including some forms of arthritis and inflammatory bowel disease, and targeting TNF has been an effective therapeutic strategy in these diseases and for cancer immunotherapy [25, 26, 239].

**Table:** 

Nomenclature	tumor necrosis factor receptor 1	tumor necrosis factor receptor 2	lymphotoxin β receptor	OX40	CD40	Fas	decoy receptor 3
Systematic nomenclature	TNFRSF1A	TNFRSF1B	TNFRSF3	TNFRSF4	TNFRSF5	TNFRSF6	TNFRSF6B
Common abbreviation	TNFR1	TNFR2	–	–	–	–	–
HGNC, UniProt	*TNFRSF1A*, P19438	*TNFRSF1B*, P20333	*LTBR*, P36941	*TNFRSF4*, P43489	*CD40*, P25942	*FAS*, P25445	*TNFRSF6B*, O95407
Adaptor proteins	TRADD	TRAF1, TRAF2, TRAF5	TRAF3, TRAF4, TRAF5	TRAF1, TRAF2, TRAF3, TRAF5	TRAF1, TRAF2, TRAF3, TRAF5, TRAF6	FADD	–
Endogenous ligands	lymphotoxin-α (*LTA*, P01374), tumour necrosis factor membrane form (*TNF*, P01375), tumour necrosis factor shed form (*TNF*, P01375)	lymphotoxin-α (*LTA*, P01374), tumour necrosis factor membrane form (*TNF*, P01375)	LIGHT (*TNFSF14*, O43557), lymphotoxin β_2_α_1_ heterotrimer (*LTALTB*, P01374Q06643)	OX-40 ligand (*TNFSF4*, P23510)	CD40 ligand (*CD40LG*, P29965)	Fas ligand (*FASLG*, P48023)	–
Ligands	–	–	–	compound 1 (Binding) (pIC_50_ 5.9) [248]	–	–	–
Comments	–	–	–	The OX40/OX40L pair is involved in late T-cell costimulatory signaling and both are transiently expressed following antigen recognition, and blocking OX40/OX40L is reported to prevent the development of disease in *in vivo* autoimmune and inflammatory disease models [279]	–	–	Decoy receptor for LIGHT (*TNFSF14*, O43557), TL1A (*TNFSF15*, O95150) and Fas ligand (*FASLG*, P48023).

**Table:** 

Nomenclature	CD27	CD30	4-1BB	death receptor 4	death receptor 5	decoy receptor 1	decoy receptor 2
Systematic nomenclature	TNFRSF7	TNFRSF8	TNFRSF9	TNFRSF10A	TNFRSF10B	TNFRSF10C	TNFRSF10D
Common abbreviation	–	–	–	DR4	DR5	–	–
HGNC, UniProt	*CD27*, P26842	*TNFRSF8*, P28908	*TNFRSF9*, Q07011	*TNFRSF10A*, O00220	*TNFRSF10B*, O14763	*TNFRSF10C*, O14798	*TNFRSF10D*, Q9UBN6
Adaptor proteins	TRAF2, SIVA	TRAF1, TRAF2, TRAF3, TRAF5	TRAF1, TRAF2, TRAF3	FADD	FADD	–	–
Endogenous ligands	CD70 (*CD70*, P32970)	CD30 ligand (*TNFSF8*, P32971)	4-1BB ligand (*TNFSF9*, P41273)	TRAIL (*TNFSF10*, P50591)	–	–	–
Endogenous agonists	–	–	–	–	TRAIL (*TNFSF10*, P50591) [306]	–	–
Agonists	–	–	–	SC-67655 [102], aponermin [57]	aponermin [57]	–	–
Antibodies	–	brentuximab vedotin (Inhibition)	–	–	tigatuzumab (Agonist) (p*K*_d_~8.5) [306]	–	–
Comments	–	–	Selective elimination of CD137 alloreactive T cells (using a single dose of an anti-CD137 antibody-drug conjugate) is proposed as a mechanism to prevent acute graft-versus-host disease (aGVHD) after allogeneic hematopoietic stem cell transplantation [87]. In monkeys however, this treatment was associated with a higher risk of reactivation and disease caused by the EBV homolog, rhesus lymphocryptovirus (RhLCV).	–	–	Decoy receptor for TRAIL (*TNFSF10*, P50591).	Decoy receptor for TRAIL (*TNFSF10*, P50591).

**Table:** 

Nomenclature	receptor activator of NF-kappa B	osteoprotegerin	death receptor 3	TWEAK receptor	TACI	BAFF receptor	herpes virus entry mediator
Systematic nomenclature	TNFRSF11A	TNFRSF11B	TNFRSF25	TNFRSF12A	TNFRSF13B	TNFRSF13C	TNFRSF14
Common abbreviation	RANK	OPG	DR3	–	–	BAFF-R	HVEM
HGNC, UniProt	*TNFRSF11A*, Q9Y6Q6	*TNFRSF11B*, O00300	*TNFRSF25*, Q93038	*TNFRSF12A*, Q9NP84	*TNFRSF13B*, O14836	*TNFRSF13C*, Q96RJ3	*TNFRSF14*, Q92956
Adaptor proteins	TRAF1, TRAF2, TRAF3, TRAF5, TRAF6	–	TRADD	TRAF1, TRAF2, TRAF3	TRAF2, TRAF5, TRAF6	TRAF3	TRAF2, TRAF3, TRAF5
Endogenous ligands	RANK ligand (*TNFSF11*, O14788)	–	TL1A (*TNFSF15*, O95150)	TWEAK (*TNFSF12*, O43508)	APRIL (*TNFSF13*, O75888), BAFF (*TNFSF13B*, Q9Y275)	BAFF (*TNFSF13B*, Q9Y275)	B and T lymphocyte attenuator (*BTLA*, Q7Z6A9), LIGHT (*TNFSF14*, O43557), lymphotoxin-α (*LTA*, P01374)
Comments	–	Acts as a decoy receptor for RANK ligand (*TNFSF11*, O14788) and possibly for TRAIL (*TNFSF10*, P50591).	The only known TNFSF ligand for DR3 is TNF-like protein 1A (TL1A) [276].	–	–	–	–

**Table:** 

Nomenclature	nerve growth factor receptor	B cell maturation antigen	glucocorticoid-induced TNF receptor	toxicity and JNK inducer
Systematic nomenclature	TNFRSF16	TNFRSF17	TNFRSF18	TNFRSF19
Common abbreviation	–	BCMA	GITR	TAJ
HGNC, UniProt	*NGFR*, P08138	*TNFRSF17* Q02223	*TNFRSF18*, Q9Y5U5	*TNFRSF19*, Q9NS68
Adaptor proteins	TRAF2, TRAF4, TRAF6	TRAF1, TRAF2, TRAF3, TRAF5, TRAF6	TRAF1, TRAF2, TRAF3, SIVA	TRAF1, TRAF2, TRAF3, TRAF5
Endogenous ligands	NGF (*NGF*, P01138) (pIC_50_ 6) [128], BDNF (*BDNF*, P23560), neurotrophin-3 (*NTF3*, P20783), neurotrophin-4 (*NTF4*, P34130)	APRIL (*TNFSF13*, O75888), BAFF (*TNFSF13B*, Q9Y275)	TL6 (*TNFSF18*, Q9UNG2)	lymphotoxinα (*LTA*, P01374)
Ligands	LM11A-31 (Binding) [184]	–	–	–
Comments	Referred to as p75, this is one of the two receptor types for the neurotrophins (factors that stimulate neuronal cell survival and differentiation), the others being the tropomyosin-related kinases (Trks). NGFR functions as a molecular switch that regulates neuronal survival and synaptic integrity, and which operates in a highly context-dependent manner. Mature neurotrophins bind preferentially to Trks and p75; proneurotrophins interact with p75 and sortilin. Unliganded and proneurotrophin-liganded p75 induce degeneration of synapses and neuronal death. In contrast, when bound to mature neurotrophins p75 forms a co-receptor with Trks that promotes neuronal survival.	–	–	Believed to be essential during embryonic development.

**Table:** 

Nomenclature	RELT	death receptor 6	TNFRSF22	TNFRSF23	ectodysplasin A2 isoform receptor	ectodysplasin 1, anhidrotic receptor
Systematic nomenclature	TNFRSF19L	TNFRSF21	–	–	TNFRS27	–
Common abbreviation	–	DR6	–	–	–	–
HGNC, UniProt	*RELT*, Q969Z4	*TNFRSF21*, O75509	–	–	*EDA2R*, Q9HAV5	*EDAR*, Q9UNE0
Adaptor proteins	TRAF1	TRADD	–	–	TRAF1, TRAF3, TRAF6	TRAF1, TRAF2, TRAF3
Endogenous ligands	–	–	–	–	ectodysplasin A2 (*EDA*, Q92838) [294]	ectodysplasin A1 (*EDA*, Q92838) [294]
Comments	Abundant in hematologic tissues. Selective receptor for TNF receptor-associated factor 1 (TRAF1). Activates the NF-κB pathway.	–	Only identified in mouse to date. A potential decoy receptor for the cytotoxic ligand TNFSF10/TRAIL. Does not contain a cytoplasmic death domain so does not induce apoptosis, and does not activate the NF-κB signalling pathway.	Only identified in mouse to date. A potential decoy receptor for the cytotoxic ligand TNFSF10/TRAIL. Does not contain a cytoplasmic death domain so does not induce apoptosis, and does not activate the NF-κB signalling pathway.	Receptor for the EDA-A2 isoform of ectodysplasin encoded by the anhidrotic ectodermal dysplasia *(EDA)* gene.	Cell surface receptor for ectodysplasin A (a morphogen involved in the development of ectodermal tissues, including skin, hair, nails, teeth, and sweat glands).

### Comments:

TNFRSF1A is preferentially activated by the sheded form of TNF ligand, whereas the membrane-bound form of TNF serves to activate TNFRSF1A and TNFRSF1B equally. The neurotrophins nerve growth factor (NGF (*NGF*, P01138)), brain-derived neurotrophic factor (BDNF (*BDNF*, P23560)), neurotrophin-3 (*NTF3*, P20783) (NTF3) and neurotrophin-4 (*NTF4*, P34130) (NTF4) are structurally unrelated to the TNF ligand superfamily but exert some of their actions through the “low affinity nerve growth factor receptor” (p75 (TNFRSF16)) as well as through the TRK family of receptor tyrosine kinases. The endogenous ligands for EDAR and EDA2R are, respectively, the membrane (Q92838[1-391]) and secreted (Q92838[160-391]) isoforms of Ectodysplasin-A (EDA, Q92838).

Further reading on Tumour necrosis factor (TNF) receptor familyBlaserH
 (2016) TNF and ROS Crosstalk in Inflammation. Trends Cell Biol
26: 249–26126791157
10.1016/j.tcb.2015.12.002CroftM
 (2024) Targeting the TNF and TNFR superfamilies in autoimmune disease and cancer. Nat Rev Drug Discov
23: 939–96139448880
10.1038/s41573-024-01053-9KallioliasGD
 (2016) TNF biology, pathogenic mechanisms and emerging therapeutic strategies. Nat Rev Rheumatol
12: 49–6226656660
10.1038/nrrheum.2015.169PMC4809675OlesenCM
 (2016) Mechanisms behind efficacy of tumor necrosis factor inhibitors in inflammatory bowel diseases. Pharmacol Ther
159: 110–926808166
10.1016/j.pharmthera.2016.01.001von KarstedtS
 (2017) Exploring the TRAILs less travelled: TRAIL in cancer biology and therapy. Nat Rev Cancer
17: 352–36628536452
10.1038/nrc.2017.28WallachD (2018) The Tumor Necrosis Factor Family: Family Conventions and Private Idiosyncrasies. Cold Spring Harb Perspect Biol
1010.1101/cshperspect.a028431PMC616981428847899XuJ (2025) The role of tumor necrosis factor receptor superfamily in cancer: insights into oncogenesis, progression, and therapeutic strategies. NPJ Precis Oncol
9: 27540770489
10.1038/s41698-025-00990-xPMC12328804
